# Structural Principles or Frequency of Use? An ERP Experiment on the Learnability of Consonant Clusters

**DOI:** 10.3389/fpsyg.2016.02005

**Published:** 2017-01-09

**Authors:** Richard Wiese, Paula Orzechowska, Phillip M. Alday, Christiane Ulbrich

**Affiliations:** ^1^Institute of German Linguistics, Philipps-Universität MarburgMarburg, Germany; ^2^Faculty of English, Adam-Mickiewicz-UniversityPoznan, Poland; ^3^Cognitive Neuroscience Laboratory, School of Psychology, Social Work and Social Policy, University of South AustraliaAdelaide, SA, Australia; ^4^Department of Linguistics, Universität KonstanzKonstanz, Germany

**Keywords:** consonant clusters, sonority, ERPs (Event-Related Potentials), learnability, Polish language, frequency

## Abstract

Phonological knowledge of a language involves knowledge about which segments can be combined under what conditions. Languages vary in the quantity and quality of licensed combinations, in particular sequences of consonants, with Polish being a language with a large inventory of such combinations. The present paper reports on a two-session experiment in which Polish-speaking adult participants learned nonce words with final consonant clusters. The aim was to study the role of two factors which potentially play a role in the learning of phonotactic structures: the phonological principle of sonority (ordering sound segments within the syllable according to their inherent loudness) and the (non-) existence as a usage-based phenomenon. EEG responses in two different time windows (adversely to behavioral responses) show linguistic processing by native speakers of Polish to be sensitive to both distinctions, in spite of the fact that Polish is rich in sonority-violating clusters. In particular, a general learning effect in terms of an N400 effect was found which was demonstrated to be different for sonority-obeying clusters than for sonority-violating clusters. Furthermore, significant interactions of formedness and session, and of existence and session, demonstrate that both factors, the sonority principle and the frequency pattern, play a role in the learning process.

## Introduction

Languages are well-known to differ in terms of combinatorial complexity of segments, especially consonants. For example, phonotactic restrictions require a word in Hawaiian to always end in a vowel (Pukui and Elbert, 1979), whereas in German strings of up to four consonant segments can be found word-finally (Seiler, 1962; Meinhold and Stock, 1980). Intervocalically, even more complex patterns are expected, with Polish permitting remarkably long combinations of up to six consonants (Rubach and Booij, 1990). By contrast, Hawaiian allows for only a single consonant in this position. Scholars in phonology have attempted to find regularities governing the combinatorial possibilities of consonants in different languages (e.g., Clements and Keyser, 1983; Rice, 1992), and to formulate general laws or principles holding for all languages. Phonotactic universals have been proposed, for instance, by Greenberg (1978) in his typological survey, but many controversies (documented in Parker, 2012) have arisen over methods and models used in predicting cluster well-/ill-formedness.

This paper contributes to the discussion on the universal and language-specific aspects of phonotactics by exploring the neurolinguistic reality of two dimensions which are potentially relevant for consonantal clusters: sonority relations as a central case of a possibly universal principle regulating phonological well-formedness of phonological structures, and clusters' existence or non-existence in a particular language instantiating language-specific differences in frequency. We report on the analysis of these two factors under the name of *formedness* and *existence*, and study the role they play in right-edge phonotactics in Polish.

Experiments on the processing of preferred clusters have drawn upon various languages and have used a number of paradigms. In their functional Magnetic Resonance (fMRI) study on English, Berent et al. (2014) found a processing advantage in terms of a reduced BOLD response in the left anterior part of Broca's area (Brodmann area 45) for clusters obeying the sonority principle. Romani and Galluzzi (2005) demonstrated an effect of sonority in the processing of Italian words by aphasic patients: a subset of patients made fewer errors in repeating words containing the preferred sonority patterns. Further evidence in favor of the relevance of both word structure and syllable structure in terms of sub-syllabic units is provided by Treiman et al. (1995), using a word game involving manipulation of phonemes and combinations of them within non-words. It was easier for participants to manipulate segments forming onset and rime clusters than other substrings. Both word onsets and syllable onsets were argued to play a role. For German, Domahs et al. (2009) studied differences in the processing of phonotactically legal and illegal clusters. This electroencephalogram (EEG) study provided evidence that listeners differentiate between phonotactically legal and illegal neologisms, even after they were detected as non-existent. These contributions, however, do not answer the question whether well-formedness and exposure play independent roles in the processing of consonant sequences.

The present experiment uses a novel learnability paradigm based on two successive EEG sessions, which allows tracing factors which facilitate or hinder the learning of phonotactics. More specifically, we can analyse how native speakers of Polish learn clusters constrained by the phonotactic principle of sonority and the (non-)existence of clusters within a period of a few days and a relatively limited exposure. Existent and non-existent clusters are used in order to study the relevance of previous exposure, while ill-formed and well-formed clusters are used to observe the role of universal principles such as sonority. Electrophysiological measures are employed, since these have been shown to be very sensitive to subtle differences in linguistic features and violations of relevant restrictions. The overall objective of this paper is to cast light on the issue of universal and language-specific factors possibly playing a role in the online processing of Polish phonotactics.

In empirical studies relating to phonology, it is possible to cross the two factors of well-formedness and existence. This is the case because phonotactic principles such as sonority may be regarded to be valid in spite of being frequently violated in existent forms (see details in Section Phonotactics). In Polish, for instance, both types of clusters are found; those which follow and those which violate the sonority principle. Users of Polish are thus familiar with both types of patterns, which raises the obvious question whether the sonority principle is a valid one. Several options are available: the principle could be true statistically, or it could be a violable principle in the sense of Optimality Theory, or it could be non-existent. In Optimality Theory (Prince and Smolensky, 1993), valid principles (“constraints”) can be violated if their violation is required by the fulfillment of other principles which are higher-ranked in the same language. In this sense, phonotactic principles contrast with well-formedness principles in other linguistic areas such as syntax where ill-formed constructions are assumed to constitute errors, at best. In addition, even for a cluster-rich language such as Polish many combinations of consonants are not attested, thus providing exemplars of non-existing clusters.

Conducting the present study on Polish and a parallel one on German (Ulbrich et al., 2016) was motivated by two premises. Firstly, as shown in Section Phonotactics, the two languages are phonotactically elaborate, and allow strings of several adjacent consonants in every word position. Secondly, the higher degree of phonotactic complexity for Polish, with numerous violations of the sonority principle is expected to provide insights into similarities and differences in the processing of the same set of non-existent clusters. A cross-linguistic study by Orzechowska and Wiese (2015) has shown that various phonotactic restrictions (e.g., place of articulation, manner of articulation, voice or length) display a different weight (for a discussion on cluster structure in terms of feature weight and ranking, see Orzechowska, 2016): sonority was demonstrated to play a much lesser role in existing Polish clusters than in German clusters. This observation raises the question whether sonority plays any role at all in the phonological processing of words for speakers of Polish.

## Phonotactics

The problem of the adequate description of clusters has been frequently addressed in the literature, cf. recent discussions by Hoole et al. (2012) and Parker (2012). Many models which have traditionally been used to distinguish between well-formed and ill-formed clusters are sonority-based, while more recent approaches emphasize the role of frequency of occurrence and exposure. Other potential principles of phonotactic organization, such as those involving (non-)identical place of articulation (see Sommerstein, 1974 and later accounts), exist, but are not studied here.

Sonority, vaguely defined as “inherent loudness of individual segment-types” (Laver, 1994, p. 156), has been applied in phonology to cast light on the structure of syllables and the existence of particular consonant combinations. In spite of the fact that the concept of sonority dates back to the 19th century work by Whitney (1865), it has repeatedly triggered numerous controversies. Sonority has been alternatively defined, with differing results, in terms of the phonetic properties pertaining to the constriction degree of the vocal tract, or, alternatively, to acoustic amplitude or audibility (cf. Sievers, 1901; Clements, 1990; or contributions in Parker, 2012). From a range of sonority hierarchies proposed, we chose the one in (1), and added affricates as a separate category. This scale was selected since it is not theory-dependent and it ensures the inclusion of all classes of consonants relevant for Polish phonology. The scale in (1) reflects the increase in opening of the articulatory tract from left to right. Except for affricates, the categories used are based on the manner features of the widely accepted IPA classification of sounds (International Phonetic Association, 2007).

(1) Sonority hierarchy
       plosive < affricate < fricative < nasal < liquid < glide < vowel

The Sonority Sequencing Generalization (Hooper, 1976; Selkirk, 1984) predicts sonority to rise from both margins of the syllable toward the syllabic peak, usually a vowel. In Spanish, for example, the structure of consonant clusters can be explained by a hierarchy given in (1). Words such as *flor* “flower” and *primo* “cousin” have a CC onset pattern, in which a more sonorous obstruent (/l/ or /r/) intervenes between the preceding less sonorous segment (/f/ or /p/) and the following vowel. However, in Polish the generalization is often violated in double initial clusters, as in /wk/ *łkać* “cry,” as much as in longer consonantal strings, e.g., /mgw/ in *mgła* “fog.” There is also a set of plateau clusters in which the member segments do not display any difference in the sonority value, as in the fricative cluster /sf/. In fact, the analysis of Polish shows that a large proportion of the cluster inventory tends to be ill-formed. In their corpus study on word-initial phonotactics, Orzechowska and Wiese (2015) analyzed 423 Polish clusters, out of which more than a half (57%) was demonstrated to violate the sonority-sequencing generalization (including 39% of plateau clusters).

Whether the existence of such exceptions discredits the relevance of sonority has been debated among phonologists. In the related approaches of usage-based phonology (cf. Bybee, 2006) and Exemplar Theory (cf. Goldinger, 1996; Pierrehumbert, 2001), phonological patterns arise through learning as such, where learning is based on frequency of occurrence in the input: learning largely consists in storing the input (exemplars), and needs only a limited amount of abstraction. Phonotactic and other structural patterns are at best epiphenomenal results of memory traces of more or less frequent exposure. In the present experiment, we examine the role of frequency by distinguishing, in a simplified binary fashion, between existent and non-existent clusters. Note that the two factors of well-formedness and existence are completely orthogonal to each other: there are non-existent but well-formed clusters, as well as existent but ill-formed ones.

When formulating hypotheses for Polish, we followed previous work suggesting that different phonological features or their combinations are preferred in different languages. For example, for the word-initial context, Orzechowska and Wiese (2015) observed that Polish favors particular patterns of place of articulation and voicing agreement, in contrast, German prefers small cluster size and a set of features pertaining to sonority. It is thus an open question whether sonority in the sense discussed above plays any role at all for Polish speakers in the processing of their language. In any case, numerous clusters exist which violate the sonority requirement. While phonotactic restrictions have mostly been applied to the unit of the syllable, there is a school of thought arguing for the expansion of such restrictions to the word domain. Therefore, following (Dziubalska-Kołaczyk, 1995; Rubach, 1996; Steriade, 1999), this work focuses on the word-final rather than the coda context. Further evidence in favor of the relevance of both word structure and syllable structure in lexical processing is provided by Treiman et al. (1995).

Polish has a large and complex consonant system, with many and manifold clusters built on these consonants in word-initial, medial, and word-final positions (see Zydorowicz et al., 2016) for a comprehensive discussion. Our list of clusters is based on the consonantal inventory given by Jassem (2003, p. 103). Out of 31 consonants provided by Jassem, only a subset was chosen for the selection of clusters. We studied clusters consisting of two segments only; see the list in Table 1 below, including clusters emerging exclusively due to morphological operations, such as /ʧp/ in *liczb* “numeral, gen. pl. (from *liczb*+*a*)” or /Iʧ/ in *walcz* “fight, imp. sg. (*walcz*+*yć*).” Note that /ʧ/, an affricate, counts as one complex segment.

**Table 1:** **Consonant clusters used for stimulus construction**.

	**Existent (EX)**	**Non-existent (NEX)**
Well-formed (WF)	ɕp, ɕʨ, fk, fʨ, jm, jp, js, lk, lm, mɕ, mʃ, mx, ɲʨ, rk, rs, sk, sp, ʃt, ʃʧ, ʧp, ʧt	fp, fʧ, lɲ, mk, nk, np, nx, ɲf, ɲp, ɲʦ, ɲʧ, ɲx, rɲ, sʦ, sʧ, ʃk, ʃʦ, ʧk, xk^a^, xp, xʧ
Ill-formed (IF)	ɕl, fn, fr, kf, kl, km, kx, mn, nr, pɲ, ps, pt, pʨ, pʧ, ʃx, tf, tr, xm, xʃ	fʃ, fx, kp, kʃ^b^, kʧ, pk, pʦ, px, sʃ, ʃf, tk, tp, ʦf, ʦʃ, ʦx, ʧf, ʧs, ʧʦ, ʧx, tx, xf

a *Instead of noxk, the stimulus noxt was used erroneously. The results for this stimulus were not used, because /xt/ is an existent cluster. The cluster /xk/ was still used in gexk and faxk*.

b *The cluster is found in Polish in the inflected word form riksz (genitive plural of riksza ‘pedicab’). However, due to its extremely rare occurrence, it was treated as non-existent in the data set*.

Phonotactic knowledge is considered to be part of phonological knowledge, containing both universal and language-specific aspects, and with an impact on the processing of clusters. Frequency of clusters has been demonstrated to have an impact on processing time: Vitevitch et al. (1997) and others reported a significant negative correlation in a repetition task between the frequency of English clusters and the reaction time to stimuli containing clusters. But similarly, effects of phonotactics have been established with respect to phonotactic knowledge: Dupoux et al. (1999) and Dupoux et al. (2001) demonstrated that Japanese listeners tend to break up consonantal clusters which do not exist in their language perceptually by “hearing” an illusory intervening vowel. These authors also showed that phonotactic knowledge is the source of these perceptual effects, operating at the prelexical stage of word processing. Furthermore, Kabak and Idsardi (2007) argued on the basis of a study with Korean listeners that language-specific restrictions on consonant sequences are based on structural units such as syllables, and not on linear sequences of consonants alone. EEG studies on the processing of phonotactics are discussed in the section to follow.

## Studies on learnability

Whether universal principles play a crucial role in phonology is one of the fundamental questions of linguistic theory. In one tradition, most notably established by Chomsky and Halle (1968), universal principles regulate the way in which a learner acquires knowledge of a language. In contrast, usage-based approaches consider the acquisition of a phonological system to be the result of a generalization over the input, resulting in probability measures; cf. (Bybee, 2001; Munson, 2001). Accordingly, frequency of the input to the language user serves as a crucial variable, perhaps as the most important one.

The two approaches share the view that learnability of structures provides a central criterion for the evaluation of specific proposals for phonological structures. From the theory of Generative Phonology (Chomsky and Halle, 1968; and others), we may derive the prediction that those structures which directly reflect universals are easy to learn. The strongest view holds that such structures do not have to be learned at all and instead are fully innate. From the perspective of usage-based phonology, we may conclude that previous experience is the basis for further learning, both at the symbolic level (e.g., existent syllables) and at the hierarchically lower levels (e.g., allowed sequences and combinations of features). Therefore, the comparison of ease or difficulty with which specific structures are learnt provides an important research tool.

Methodologically, the present experiment on learnability compares the behavioral and neurophysiological reactions (over time) to minimally different structures; an approach that has been used in studies on artificial grammar learning (see Gómez and Gerken, 2000, in phonology, Mueller et al., 2005, in other areas of grammar). The fundamental hypothesis of the approach is that different structures display different degrees of learnability that can be measured. Furthermore, the EEG paradigm in the study of learnability allows to test whether changes in neural reactions to particular linguistic features over a pre-specified time period (2–4 days, in the present case) can be found.

The study of learnability in general has been based on the principle that the amount of exposure to a new stimulus determines the degree of its mastery; however, short exposure has been claimed to suffice in the learning process even for infants (Gomez and Gerken, 1999). Learnability of novel CVC syllables after an auditory experience of several seconds was tested in infants (Chambers et al., 2003) and after a several-time repetition of target items in adults (Onishi et al., 2002). In other studies, relevant measurements were conducted after extended exposure, ranging from 35 min (Bahlmann et al., 2008) to 50 h spread over 5 weeks (McCandliss et al., 1997). Previous research on learning phonotactic patterns ranges from studies on the acquisition of phonology (Jusczyk et al., 1994) and grammar to works on the learning of second language features (Redford, 2008). Learning of grammar has been tested on the basis of (il)legal phonotactics (Bahlmann et al., 2008) and its processing (Rossi et al., 2011).

Novelty of linguistic items has been demonstrated to evoke an N400 effect, i.e., negativity after about 400 ms of detection, cf. Kutas and Federmeier (2011) for a review. In EEG studies of phonotactic principles, Domahs et al. (2009) and Moore-Cantwell et al. (2013) found an early negativity effect (N400) for existent vs. non-existent (novel) monosyllabic strings, demonstrating the role of lexical knowledge. In addition, a later positivity effect (LPC) for those nonce items which violated a specific phonotactic principle disallowing a /sC_1_VC_1_/ structure was found by Domahs et al. (2009). (In both English and German, words of this type, such as /spip/, do not exist or are very rare.) For Moore-Cantwell et al. (2013), the phonotactic phenomenon studied was voicing agreement in a structure of the type C_1_VC_2_V. The perceptual illusory vowel referred to with respect to the perception of clusters by Japanese listeners above was confirmed by an EEG study by Dehaene-Lambertz et al. (2000): a mismatch negativity reaction to deviating clusters was obtained for French listeners, but not for Japanese listeners.

Since the main consideration is whether specific linguistic structures are learned more easily than others over the same period of time, the basic approach adopted here is one of learnability. In other words, the ease or difficulty with which participants acquire new phonological structures constitutes the main criterion for the data analysis. Our understanding of the process of learning is similar to that in studies based on *artificial grammar learning;* cf. (Bahlmann et al., 2008; Friederici et al., 2006); as in these studies, we assume that the relative ease or difficulty with which linguistic items based on particular constructions can be learned provide insight into the mental representation of the constructions compared.

## Hypotheses

The experiment introduced nonce words as names for unusual physical objects. All stimuli were presented eight times during the course of the experiment, twice during the first EEG session (pre-learning, EEG-1), four times during an online training, and twice again during the subsequent second EEG session (post-learning, EEG-2). The design of the experiment allows for making comparisons not only within each experimental session but also between sessions EEG-1 and EEG-2. Since behavioral data is generally less sensitive with respect to subtle linguistic properties, we formulate separate sets of hypotheses on the learnability of clusters for the behavioral and neural reactions (accuracy and event-related potential (ERP) responses, respectively).

Predictions for the behavioral data are as follows:

1. Session: correctness rates increase from session EEG-1 to session EEG-2.
2. Existent vs. non-existent clusters: accuracy for existent clusters is higher than for non-existent ones within EEG-1 and EEG-2.
3. Well-formed vs. ill-formed clusters: well-formed clusters do not display increased accuracy compared to ill-formed clusters in either EEG-1 or EEG-2.

Predictions for the ERP responses *within* a single EEG session are as follows:

4. Existent vs. non-existent clusters: differences between the processing of existent and non-existent clusters are found. More specifically, non-existent clusters are novel linguistic items for which an N400 effect is expected.
5. Well-formed vs. ill-formed clusters: generally, we expect *no* significant differences for the processing of well-formed and ill-formed clusters with speakers of Polish. However, due to the possibly universal status of sonority, reactions to sonority violations may occur even in Polish, especially in the existence-sonority interaction, for which a late positive component (LPC) for non-existent ill-formed clusters is expected.

Predictions for the ERP responses *across* EEG sessions are as follows:

6. Existent vs. non-existent clusters: the difference between these clusters in terms of an N400 decreases from EEG-1 to EEG-2.
7. Well-formed vs. ill-formed clusters: late positivity effects are expected to decrease from EEG-1 to EEG-2.

## Experiment

### Participants

The experiment took place in the *Center for Speech and Language Processing* at the Faculty of English, Adam Mickiewicz University, Poznań. Participants were recruited at the university, with the majority of them coming from the region of Western Poland. Twenty-seven participants (13 women) took part in the experiment, out of which 4 had to be excluded for not correctly completing all sessions. Their age ranged from 18 to 30 years (with a mean of 23.5). All the participants were brought up in a monolingual context, right-handed and reported no vision or hearing problems. The participants all gave informed consent for participation and were financially compensated for their contribution.

### Materials

#### Stimulus construction

When preparing the cluster list, all possible CC strings were generated automatically on the basis of the set of consonants found for Polish. To ensure that the same set of clusters could be tested in the Polish and in an identical German experiment (see Section Discussion), we eliminated all the combinations in which the final sonorant can be syllabic in German, e.g., /ml/, /tn/, or /fr/, and /n/+fricative sequences in which the nasal is realized as a nasalized labio-velar glide in Polish, e.g., *sens* /se$\tilde{w}$s/ “sense.” In terms of phonetic identity of segments in Polish and German, some compromise was necessary; for instance, the Polish prepalatal /ɕ/ was considered similar enough to the German palatal /ç/.

The remaining clusters were further classified into those which (a) obey or disobey the sonority sequencing generalization, and (b) are existent or non-existent, but could possibly be introduced into Polish on the basis of their segmental composition. Plateau clusters according to the sonority scale in (1) were considered to be ill-formed. As a result, we arrived at four groups of clusters: existent-well-formed (EX-WF), existent-ill-formed (EX-IF), non-existent-well-formed (NEX-WF), and non-existent-ill-formed (NEX-IF). The set of clusters used in the experiment is given in Table 1.

The maximum number of clusters to be found within all four groups was 21. For the EX-IF group, only 19 items were available. Therefore, some existent clusters were used twice, in combination with prefixes in which the vowels *e, a, o* were exchanged. To ensure maximal similarity between clusters, the existent clusters were matched with the non-existent ones according to two criteria: phonetic similarity in terms of places and manners of articulation, following the IPA description (International Phonetic Association, 2007). Therefore, some of the given clusters emerging due to morphological operations or with very low type and token frequency, e.g., /fn/, /km/, /kf/, /nr/ as in *hafn* “hafnium,” *flegm* “phlegm” (genitive plural), *strzykw* “sea cucumber” (genitive plural), *henr* “henry” (a physical unit), had to be used. Since there are no syllabic consonants in Polish, obstruent+sonorant clusters such as /pɲ, kl, fn/ are legitimate tautosyllabic clusters. In Appendix 1, we present a list of existent Polish words containing the clusters listed in Table 1.

Stimuli were monosyllabic nonce words containing the final CC clusters listed in Table 1. The structure of each nonce word was: CV-sequence + CC-cluster. In order to increase the number of items to be used, the critical clusters were preceded by three different CVs, namely *ge, fa, no*, all of which are acceptable and unmarked in Polish. The three contexts *ge, fa, no* allowed for the presentation of each cluster in three different nonce words, as in /gekʧ/, /fakʧ/ and /nokʧ/.

A phonetically trained female speaker of Polish, coming from the west-central Poland region, spoke each nonce word at a normal speech rate. Each item was recorded in a 16bit resolution and a sampling rate of 44.1 kHz. To avoid unnaturally careful pronunciation of the clusters, to be expected since some of the clusters were articulatorily demanding, and to ensure authentic but clear pronunciation, stimuli were recorded under the supervision of a phonetician.

The number of critical clusters and nonce words used within a single condition was thus 21 (nonce words with target clusters) × 2 (existent vs. non-existent) × 2 (well-formed vs. ill-formed) × 3 (CV contexts), resulting in 252 nonce words. These auditory stimuli were presented as names of unknown objects, in particular of exotic animals, plants, or unknown artifacts. For this purpose, 252 pictures of such objects were collected from various sources on the internet. They were selected on the basis of the unfamiliarity and the different object categories presented and assigned to the verbal stimulus items at random. Pictures were standardized in terms of size (425 × 425 pixels, 15 × 15 cm) and presented on a black screen. The task for the participant in each trial was to learn a new name for an unusual object, which constitutes an ecologically valid verbal task of learning a new vocabulary item. This task also ensured that participants would not focus explicitly on the phonotactic properties of the stimuli.

#### Phonetic analysis

Stimuli were cut from the recordings at the beginning and at the end of the word using the Amadeus Pro software (HairerSoft, Kenilworth, UK; Version 2.1, 1523). In order to see whether items from the four conditions differed in terms of phonetic parameters, they were checked *post-hoc* for three basic acoustic properties, namely duration, fundamental frequency (F0) and amplitude. Table 2 presents a summary of the results.

**Table 2:** **Phonetic parameters of the stimuli; means and standard deviations for pitch (Hz), duration (sec), and amplitude (dB SPL)**.

**Stimulus type**	**F0 (Hz)**	**Duration (sec)**	**Amplitude (dB SPL)**
WF-EX (*n* = 63)	210.4 (±12.9)	0.71 (±0.05)	52.4 (±4.26)
WF-NEX (*n* = 62)^a^	210.1 (±11.2)	0.85 (±0.08)	50.9 (±4.08)
IF-EX (*n* = 63)	229.5 (±15.8)	0.76 (±0.11)	49.8 (±4.40)
IF-NEX (*n* = 63)	227.2 (±12.4)	0.90 (±0.09)	48.0 (±3.71)

a *See footnote b in Table 1*.

As shown, the stimulus items differed from each other in terms of the three acoustic parameters. As far as mean fundamental frequency is concerned, the well-formed items had lower F0-values than the ill-formed ones for both the existent and non-existent groups. However, the mean pitch differences of 17–18 Hz, corresponding to 1.3–1.5 semitones, are considered to be below the perceptual threshold. Nooteboom (1997) and Hart et al. (1990, p. 29) argued that a difference of 3 semitones is needed for pitch to be discriminable by humans, a lower threshold of 1.5 semitones, argued for by Rietveld and Gussenhoven (1985, p. 304) is barely reached in the differences of the analyzed stimuli.

All four groups of stimuli were distinct from each other in terms of duration. Both non-existence and ill-formedness added to the length of the items. However, these differences are not unexpected. First, frequent words have been demonstrated to be shorter than infrequent ones; (cf. Wright, 1979, or Gahl and Garnsey, 2004). Second, well-formed clusters cannot be expected to be phonetically identical to ill-formed ones, and may possibly be preferred cross-linguistically precisely because they conform to demands of articulatory ease. Differences found for duration were small, however, with the non-existent items being about 0.140 s. (16%) longer than the existent ones. The impact of such differences is unclear as the participants start to process the stimuli from their onset, while their length can be fully evaluated only at the stimulus offset. As for amplitude, the WF-EX items differ from the IF-EX and IF-NEX items, and the WF-NEX items differ from the IF-NEX ones. The differences found ranged from 2.5 to 4.4 dB, and display lower amplitudes for the non-existent and ill-formed items. In order to evaluate the role of the three phonetic parameters, we provide information on a full statistical model which includes these phonetic parameters as covariates in the model (see Appendices 3, 4).

### Procedure

#### Overall experimental design

The study consisted of two experimental sessions involving learning, with an intervening online training. The over-all design of the experiment is given in Table 3.

**Table 3:** **Design of the learnability paradigm**.

**Design**	**Phases**	**Time**
Session 1: EEG-1	Stimulus-presentation	day 1
	Response-elicitation	
Online training	Stimulus-presentation	day 2 (or 3)
	Response-elicitation	
Session 2: EEG-2	Stimulus-presentation	day 3 (or 4)
	Response-elicitation	

An EEG paradigm was chosen because of the high temporal resolution in the recording the brain activity online and in a non-invasive manner. In each session, participants were presented with nonce words and pictures of corresponding objects. The nonce words contained the total of 84 clusters, 21 representing each condition (well-formed vs. ill-formed, existent vs. non-existent; see Table 1). During session 1, participants were exposed twice to the critical stimuli while the ERPs and behavioral responses were recorded. The same word-picture pairs were tested during the EEG recording in session 2, following the online training. Instructions as to the procedure were given prior to the session, and further instruction, if necessary, following the training. During training, they were exposed to a set of 21 practice trials, i.e., word-picture pairs. Each experimental session took approximately 60 min, including training and breaks. The main goal was to expose the participants to the same data set, which totalled up to 8 repetitions of each item (2 repetitions in 2 EEG sessions in addition to 4 repetitions during the online practice sessions).

Participants were seated in a sound-attenuated and dimly lit cabin in front of a screen. Recordings in each session were divided into 12 blocks of 21 word-picture pairs, i.e., a total of 252 items. In order to present a balanced number of items which could be remembered after the first exposure, 21 nonce words together with corresponding pictures were used in each block. After every block, the participants were allowed to take a short break. A longer break took place after the 6th block.

Each subject was presented with the same set of 252 words and pictures. To ensure that participants did not inform each other about details of the experiment, in particular the word-picture pairs and their ordering, each subject was provided with a different version of the experiment. Randomizations were performed over the word-picture mappings, and the ordering of trials within a block, resulting in 12 different versions of the experimental material. Additionally, to avoid a handedness bias, the 12 blocks of 21 items were used once with the correct response assigned to the right joystick button, and once to the left button. The same version of the material (ordering of trials, word-picture pairs, handedness) was assigned to the same participant in sessions 1 and 2. In the online training, the word-picture matching was also identical to that in the two EEG sessions.

Each block had a twofold structure: a stimulus-presentation phase and a response-elicitation phase. In the first phase, the participants were presented with the nonce words and pictures matching them, and were instructed to remember as many pairs as possible. The second phase consisted in eliciting responses for the pairs presented earlier.

In the presentation phase, each trial started with the auditory presentation, via loudspeakers, of a nonce word with a target cluster, e.g., *fak*ʧ corresponding to an exotic fruit. With the onset of each word, a fixation star appeared on the screen for 1500 ms. The length of the auditory stimuli varied from 600 to 1200 ms, with an average of 800 ms. Next, the participants were exposed to the corresponding picture for 1500 ms, followed by 1500 ms of blank screen before the next trial. The same procedure was repeated for every pair in one block (21 times). Each block was initiated by a synthesized sine wave (340 Hz) of 500 ms duration.

In the elicitation phase, the participants were exposed to the same set of 21 word-picture pairs. For half of the items, the matching between words and pictures varied from that in the presentation phase, e.g., *fak*ʧ corresponding to an unusual type of fish. The order of stimulus presentation was the same as in the first phase. The presentation of the picture was followed by a question mark on the screen, with a timeout of 2000 ms. During this time, the participants were expected to decide whether the matching of the word to the picture corresponded to that introduced in the presentation phase by pressing a “yes-no” joystick button (left-right counterbalanced across participants). After the response, the screen remained blank for 1500 ms. During the period from the offset of the visual stimulus to the onset of the auditory stimulus in the next trial, participants were allowed to blink and rest their eyes.

#### EEG recordings

The EEG was recorded by means of 27 Ag-AgCl electrodes with the AFz electrode serving as ground electrode. The reference electrode was located at the left mastoid. EEGs were re-referenced off-line to both the left and the right mastoid. In order to control for eye-movement artifacts, electrodes fixed above and below the participants' left eye as well as electrodes placed at the lateral canthus of both eyes (electrooculogram, EOG) recorded the vertical and horizontal eye movements respectively. Impedances of electrodes were kept below 5 kΩ. EEG and EOG measurements were continuously recorded by a BrainAmp amplifier (Brain Products, Gilching, Germany) and digitized at a rate of 500 Hz. Results were filtered off-line with a FIR zero-phase bandpass filter from 0.16 to 30 Hz (edges of passband). Trial epochs were generated from −200 ms to +1200 ms, time locked to the peak of the vocalic nucleus.^1^ No baseline correction was used because none of the analyses required zero-mean, the early exogenous components overlapped sufficiently, and baseline correction can potentially introduce activity from the pre-stimulus period (cf. Maess et al., 2016). Trials with artifacts were threshold-rejected automatically (average exceeds 40 μV in a 200 ms sliding window within the entire epoch). Rejections were relatively few on average and did not vary systematically between conditions (see Table 4).

**Table 4:** **Average Number of Trials Remaining per Subject After Artifact Rejection**.

**Session**	**Existence**	**formedness**	**remaining trials (mean)**	**sd**
1	Existent	ill	59.0	4.0
1	Existent	well	57.8	4.8
1	Non-existent	ill	57.4	4.8
1	Non-existent	well	58.9	3.7
2	Existent	ill	59.9	3.3
2	Existent	well	59.8	4.2
2	Non-existent	ill	59.3	3.4
2	Non-existent	well	59.6	4.4

*There was a total of 63 items per condition and session*.

For the analysis of ERPs, data from the response-evaluation phase in both sessions were used. These can be considered more reliable than ones from the stimulus-presentation phase, as the participants heard the stimuli for the second time within each session and because the test subjects were actively engaged due to the task. The stimulus-presentation phase in the parallel experiment with German speakers (Ulbrich et al., 2016) was used to determine relevant time windows to be used for the present analysis, one from 450 to 550 ms and one from 700 to 1050 ms.^2^ These values were thus chosen independently of the data in the present experiment, but not in an arbitrary manner, as they ensure a direct comparison between the data from the two languages.

ERPs to be analyzed were time-locked to the peak of vowels in each stimulus, because information on the nature of the consonants to follow may be available from this point onwards. The peak of the vocalic nucleus was defined as the intensity peak of the vowel in each stimulus. These intensity peaks were computed with the help of a Praat script (de Jong and Wempe, 2009).

#### Online training

The online training was made possible by the internet-based learning and teaching platform of the University of Marburg. In this training, the participants were provided with the same word-picture pairs as in EEG-1 and EEG-2, and were instructed not to do the online training right after the first or just before the second EEG measurement. Similar to the EEG sessions, each online test was divided into 6 blocks. Each block consisted of a stimulus-presentation (learning or training) phase, during which the participants were exposed to 42 target word-picture pairs (presented one at a time), and a response-elicitation (testing) phase, which consisted in testing the word-picture pairs just learned.

During the presentation phase, pictures and the corresponding auditory stimuli were presented. After exposure to one trial, the participants continued by pressing the button “next.” Four sets of tests (A, B, C, D) were devised, differing with respect to word-picture pairs and their order. The elicitation phase was based on the same procedure; however, the matching between words and pictures was changed for half of the pairs. Participants were requested to decide whether the matching was correct or not, by clicking the “yes” or “no” button, following the question below the picture presented, i.e., “Does the word match the object?” Each of the 6 blocks was worked on twice (in each phase), which increased the participants' exposure to the items. During the presentation and elicitation phase, each stimulus was thus heard four times. Participants had the possibility of accessing their results after the completion of the online training.

## Results

### Behavioural data

**Figure 1:**
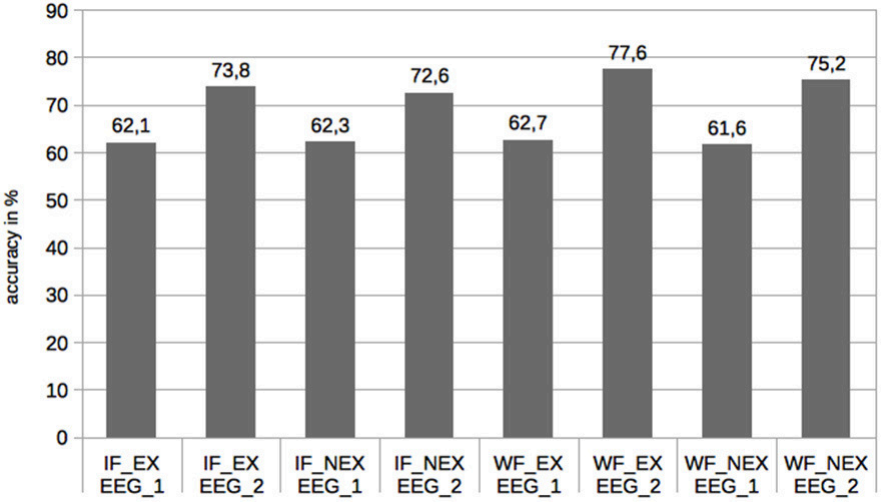
**Correctness rates in relation to conditions**.

The results for the correctness rates are given in Figure 1. The mean accuracy increased from EEG-1 to EEG-2 for all the conditions with a range from 10.3 to 14.9 percentage points, demonstrating that learning was successful.

In the analysis of these behavioral data (accuracies in the elicitation phase), we performed a logistic mixed-effects model, including sonority with 2 levels (WF and IF), existence with 2 levels (EX and NEX), and session with 2 levels (EEG-1 and EEG-2) as fixed factors. Due to the partial malfunctioning of the “yes”-“no”-button-box, data from only 22 participants could be analyzed. The results are presented in Table 5, and a full model summary is provided in Appendix 2.

**Table 5:** **Logistic mixed-effects model for accuracy; Analysis of Deviance (Type II Wald χ^2^-tests)**.

	**χ^2^**	**Df**	**Pr(> χ^2^)**	
Session	198.9165	1	<2e-16	^***^
Existence	1.9720	1	0.16023	
Formedness	2.3095	1	0.12858	
Session:existence	0.9559	1	0.32822	
Session:formedness	3.8290	1	0.05037	.
Existence:formedness	0.5846	1	0.44453	
Session:existence:formedness	0.0061	1	0.93795	

Significance Codes: “^***^” 0.001, “^.^” 0.1.

The analysis revealed a highly significant effect for the session variable only. Since the main effects for sonority and existence as well as all interactions did not achieve statistical significance, their role in correctness judgements cannot be established. In summary, while correctness rates, not surprisingly, increased from session 1 to session 2, they cannot be shown to be sensitive to the main experimental variables of formedness and existence. For the behavioral measure of accuracy, hypotheses 1 and 3 can be confirmed, while hypotheses 2 can not.

### EEG data

Data from 23 participants was used in the analysis of the EEG responses. Material to be analyzed thus comprised 8 conditions ^*^ 10 electrodes ^*^ 63 items ^*^ 23 participants, equalling 131 040 observations, which dropped to 108 500 when artefactual trials are excluded (see Table 4 for more detailed rejection statistics).

**Figure 2:**
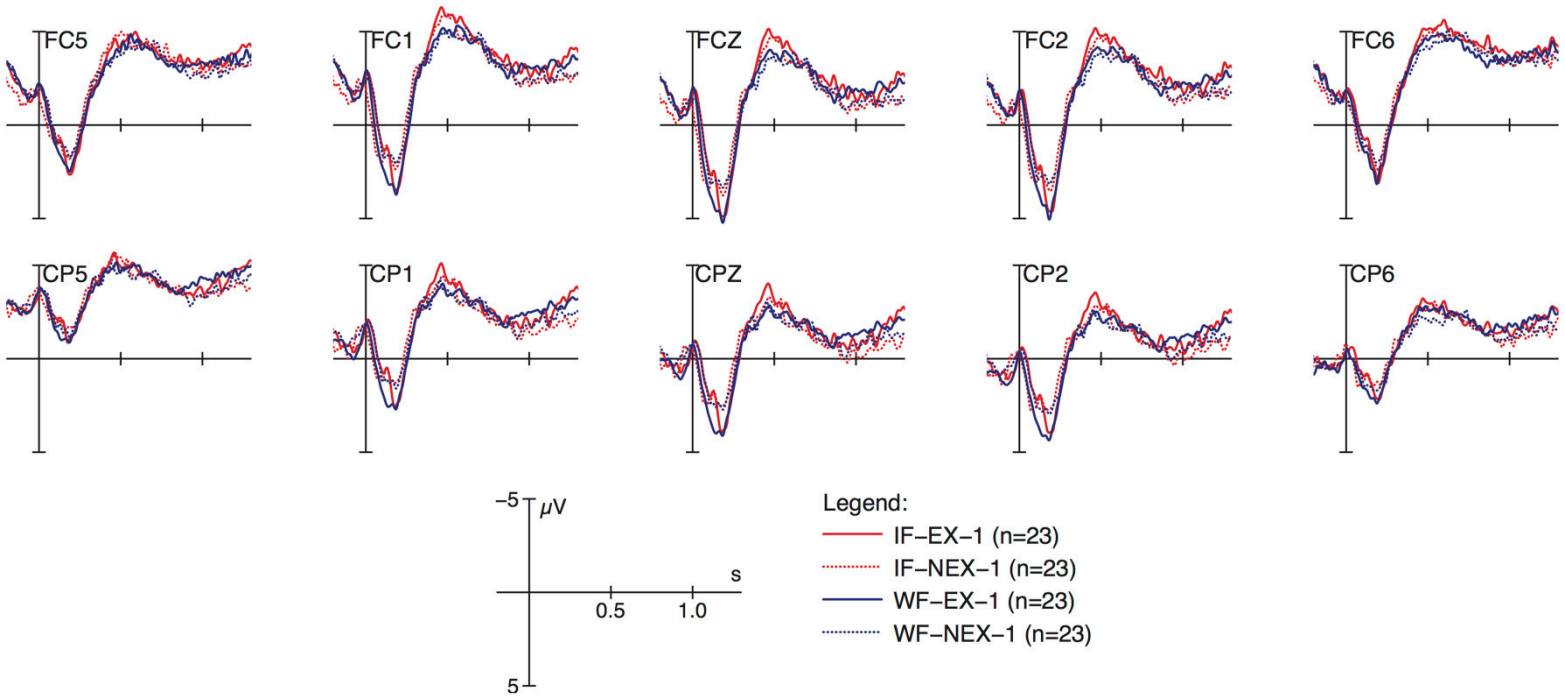
**ERP responses to stimuli at anterior (top) and posterior (bottom) electrodes; data from response-elicitation phase, session 1**.

**Figure 3:**
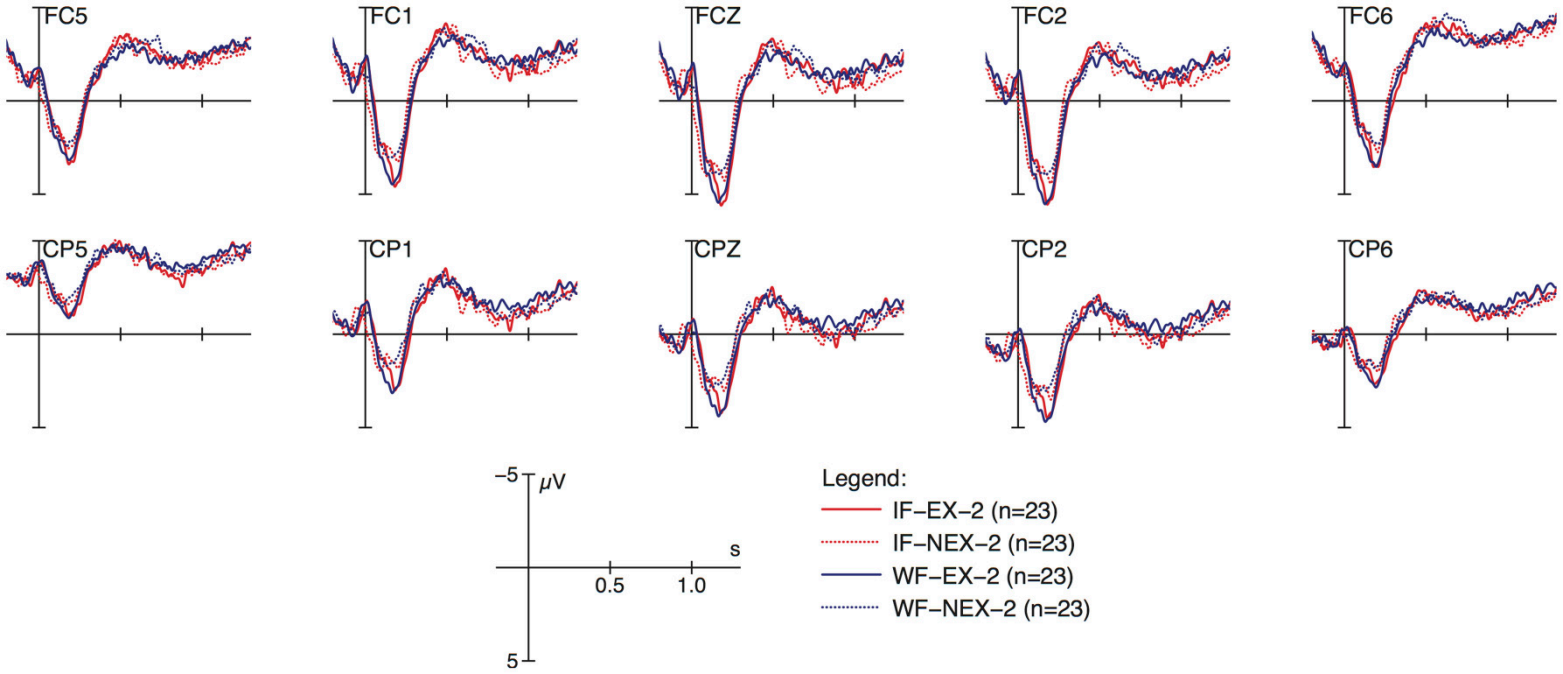
**ERP responses to stimuli at anterior (top) and posterior (bottom) electrodes; data from response-elicitation phase, session 2**.

Figures 2, 3 display grand average ERP responses from the response-elicitation phase of session 1 and 2, respectively, with the peak of vocalic nuclei as zero onsets. Ten anterior and posterior electrodes selected to constitute regions of interest (ROI, see below) are displayed with separate graphs for the four experimental conditions of formedness (ill-formed i, well-formed w) and existence (existent e, non-existent n, response-elicitation t, session1 f, session 2 s). As shown in Figure 2, responses to ill-formed items (red lines) show increased negativity compared to well-formed items (blue lines) in the time-window around 500 ms post onset, more pronounced at anterior electrodes. Furthermore, non-existent items (dotted lines) show a positive-going response around 900 ms. These differences are not pronounced in the graphs of Figure 3, where obvious differences between conditions are not apparent by visual inspection.

The results of EEG measurements were analyzed by means of a linear mixed model (Baayen et al., 2008; Bates et al., 2015), with the following four fixed factors of interest and two random factors (subject and item): session (EEG 1 and EEG 2), region of interest (anterior, corresponding to electrodes FC1, FC2, FCz, FC5, FC6, and posterior, corresponding to electrodes CP1, CP2, CPz; CP5, CP6), existence (existent and non-existent), formedness (well-formed and ill-formed). Mean EEG amplitude was used as the dependent variable. In addition to the factors of interest, the phonetic parameters pitch (Hz), duration (ms), intensity (dB) were included as nuisance parameters on the basis of the results presented in 5.3.2 in order to control for possible confounds (cf. Sassenhagen and Alday, 2016). Since the full models are quite complex, their full summaries are in the Appendices 2, 3, while in the main text, we present selected Wald Type-II Chi-squared tests, which provide an ANOVA-like summary of effects. Again, due to the large number of parameters in the model (4 effects of interest and 3 nuisance parameters have potentially 7 way interactions), we only present effects of interest in the Wald Type-II summaries. Although Wald tests can be somewhat anti-conservative, they provide a convenient summary of effects.

To test whether ill-formed clusters are learned differently from well-formed ones, and whether existent clusters are learned differently from non-existent ones, the interactions between session and formedness/existence should provide the crucial information: hypotheses 6–8 (Section Hypotheses) predict that—even if other factors are relevant—interactions of session and formedness, and of session and existence should contribute to the overall model. We tested this assumption by means of a linear mixed model in each time window with the factors just enumerated. Results for the two time windows mentioned are presented in the following sections.

#### 450–550 ms

**Figure 4:**
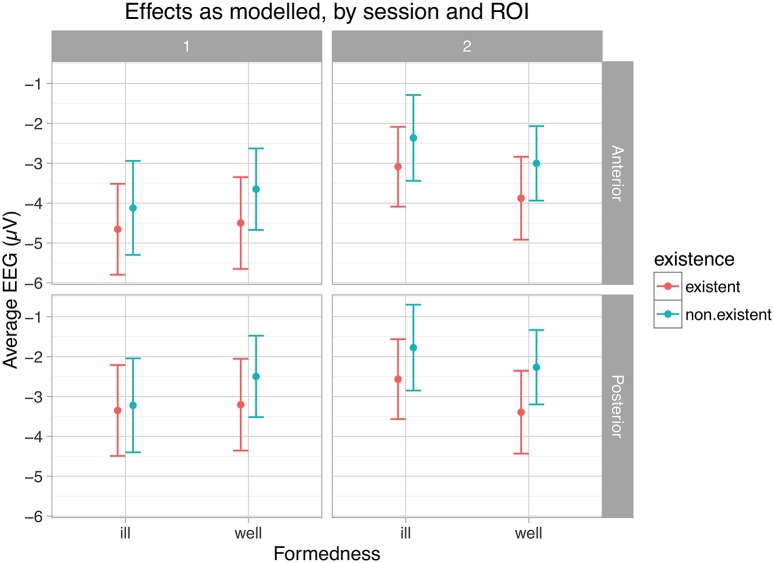
**Main effects and interactions for the time window 450–550 ms**. EEG-sessions are denoted by 1 and 2, ROIs by Anterior and Posterior.

Table 6 and Figure 4 present the results of the statistical analysis for the first time window. As justified above, we concentrate on the main experimental factors and particularly on the interactions of both formedness and existence with session.

**Table 6:** **Linear mixed model fit by maximum likelihood for the factors studied (without acoustic factors); main effects and their interactions in time-window 450–550 ms; Analysis of Deviance (Type II Wald χ^**2**^-tests)**.

	**χ^2^**	**Df**	***p***	**Sign. level**
Session	11.1519	1	0.0008394	^***^
Roi	419.6473	1	<2.2e-16	^***^
Existence	2.3460	1	0.1256082	
Formedness	2.4915	1	0.1144641	
Session:roi	53.1963	1	3.018e-13	^***^
Session:existence	0.3753	1	0.5401537	
Roi:existence	2.1573	1	0.1418936	
Session:formedness	6.5098	1	0.0107281	^*^
Roi:formedness	3.2580	1	0.0710742	.
Existence:formedness	0.2976	1	0.5853889	
Session:roi:existence	1.2374	1	0.2659641	
Session:roi:formedness	0.3121	1	0.5763748	
Session:existence:formedness	1.4656	1	0.2260411	
Roi:existence:formedness	1.5001	1	0.2206562	
Session:roi:existence:formedness	0.0183	1	0.8924935	

Significance Codes: “^***^” 0.001, “^*^” 0.05, “^.^” 0.1.

As shown in this table, the main effects of session, ROIs and their interaction contribute significantly to the model. In particular, neural responses reduce in negativity from the first EEG session to the second, and are more distinct in the anterior region than in the posterior (see Figure 2 and full model summary in Appendix 3). Most importantly however, the interaction between session and formedness is significant in this time window ($\chi_{\left(1\right)}^{2}=$ 6.5098, *p* ≤ 0.01). In contrast, there is no main effect for formedness and existence.

**Figure 5:**
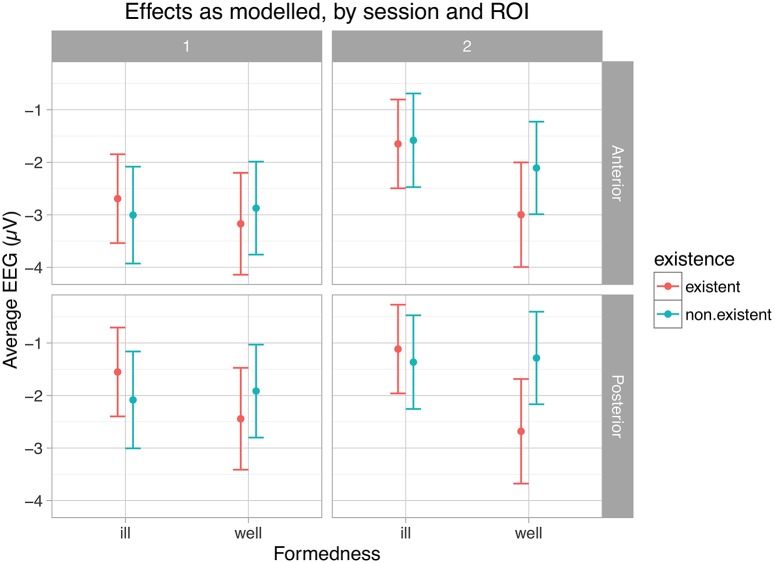
**Main effects and interactions for the time window 700–1050 ms**. EEG-sessions are denoted by 1 and 2, ROIs by Anterior and Posterior.

Figure 4 (just as Figure 5 below) illustrates these types of differences found for the experimental factors, by presenting and comparing overall means and corresponding confidence intervals (95%) of ERPs as modeled within the given time windows with respect to crucial conditions (session, ROI, formedness, existence). As shown here, ill-formed and well-formed clusters show the same degree of negativity in session 1, whereas they differ in session 2. In other words, negativity for ill-formed clusters is less pronounced in session 2 compared to session 1; but for well-formed clusters this is not the case. Thus, ill-formed clusters show an effect of learning (reduction in negativity), but well-formed clusters do not. In contrast, no such learning effect was observed for existence ($\chi_{\left(1\right)}^{2}$ = 0.38, *p* ≤ 0.54). Despite the tendency visible in Figure 4, there was no three-way interaction effect between session, formedness and existence ($\chi_{\left(1\right)}^{2}$ = 1.47, *p* ≤ 0.23).

#### 700–1050 ms

The second time window applied in the analysis was 700–1050 ms post-nuclear onset, for which we observed several significant effects and interactions as illustrated in Table 7 and Figure 5. Since detection of a later time window was generally associated with positivity (i.e., LPC / P600), in the analysis to follow we interpreted the effects found around 700–1050 ms as positivity. Note that the peak of the vocalic nucleus was defined as onset, which means that complete information on the nature of the consonant cluster is available only somewhat later, at a point which cannot be specified precisely, but presumably within the P600 period.

**Table 7:** **Linear mixed model fit by maximum likelihood for the factors studied; main effects and their interactions in time-window 700–1050 ms; Analysis of Deviance (Type II Wald χ^2^-tests)**.

	**χ^2^**	**Df**	***p***	**Sign. level**
Session	5.3865	1	0.020293	^*^
Roi	724.8985	1	<2.2e-16	^***^
Existence	3.5762	1	0.058613	.
Formedness	5.2410	1	0.022060	^*^
Session:roi	21.8537	1	2.943e-06	^***^
Session:existence	5.1535	1	0.023200	^*^
Roi:existence	0.3703	1	0.542836	
Session:formedness	5.3824	1	0.020341	^*^
Roi:formedness	0.1164	1	0.732920	
Existence:formedness	9.2795	1	0.002317	^**^
Session:roi:existence	1.2512	1	0.263315	
Session:roi:formedness	1.1135	1	0.291320	
Session:existence:formedness	0.0000	1	0.994863	
Roi:existence:formedness	5.4697	1	0.019349	^*^
Session:roi:existence:formedness	1.6911	1	0.193455	

Significance Codes: “^***^” 0.001, “^**^” 0.01, “^*^” 0.05, “^.^” 0.1.

We found main effects for session, ROI and formedness in this time window. As in the early time window studied, the interaction of session and formedness turned out to be significant, but additionally, the interaction of session and existence was significant as well. That is, for both formedness and existence a learning effect was observed. The three-way interaction of ROI with formedness and existence was significant as well. Appendix 4 presents a full model summary.

Figure 5 shows this pattern of results. Ill-formed clusters lead to less negative, i.e., more positive responses then well-formed ones, an effect which is only strengthened by learning. Similarly, the positivity for non-existent clusters was also enhanced by learning. The three-way interaction between ROI, formedness and existence can be seen in the swapping of the absolute rankings of existence across ROIs and formedness, e.g., existent ill-formed clusters were more positive posteriorly across both sessions, while non-existent well-formed clusters were more positive posteriorly and anteriorly across sessions.

In summary, we found the following sets of significant results:

1. accuracy (increase from session 1 to session 2)
2. EEG effects, early time window: session (reduced negativity for session 2), interaction session-ROI (stronger anterior negativity in session1, but not 2), and session-formedness (reduced negativity for ill-formed clusters in session 2)
3. EEG effects, late time window: session (increased positivity for session 2), formedness (well-formed clusters elicited a larger positivity), interaction session-ROI (the positivity for session 2 was less pronounced posteriorly), interaction session-existence (increased positivity for non-existent clusters in session 2), interaction session-formedness (increased positivity for well-formed clusters in session 2), interaction existence-formedness (well-formed existent clusters show the smallest positivity across ROIs and sessions), interaction ROI-existence-formedness (positivity strongest posteriorly).

## Discussion

Ever since Trubetzkoy (1967) identified the “demarcative function” of phonotactic patterns, it has been stressed that phonotactics serves an important function in marking boundaries of linguistic units, particularly of words. This is why an account of clusters and their role in a language is central to a proper account of word processing. The processing can be based on phonotactic, possibly universal, principles (in our case, sonority) and/or on usage-based principles, in this case on the amount of exposure, i.e., (non-)existence, as the central concept.

The most important results are those dealing with the interaction of session with both formedness and existence. The present study has provided evidence that sonority and existence play a role in the processing of phonotactics by native speakers of Polish, and has thus demonstrated that two factors, which have been discussed as critical for the description of consonantal clusters, have a direct influence on the learning of such clusters. Both formedness and existence shape the neural reactions to newly learned vocabulary items containing such clusters over a 2–3-day learning span. These results also show that the design of the experiment allows to trace a learning process for linguistic structures. The results of the behavioral and the electrophysiological measures coincide w. r. t. the learning effect from session 1 to session 2, while only the electrophysiological measures proved to be sensitive enough to respond to the crucial conditions of sonority and existence.

We may attribute the differences found in the time-course of reactions to existence and formedness to a difference in the status of the two factors studied: the processing of consonantal clusters with respect to sonority relations may be based on signal properties, i.e., local features to be detected in the complex signal. In this process, sonority may operate as a special filter which allows for the fast and relatively effortless perception of relevant clusters. Whether this function is based on phonetic properties alone or on a deep-rooted linguistic universal (as argued by Berent et al., 2008, 2014) remains an open question. Therefore, a reaction based on this structural property is expected. In contrast, existence or non-existence of a cluster is a property requiring access to some sort of repertoire of phonological objects, often called phonological lexicon; see, e.g., (Westbury et al., 2002). For this reason, it is not surprising that formedness shows an early effect, but existence does not.

We consider the first time window 450–550 ms to reflect an N400 component. This component has been shown to increase in the processing of nonce words, pseudowords or more generally neologisms (Bentin et al., 1999; Domahs et al., 2009) vs. existing words. The latter observed a non-significant difference between ill-formed nonce-words and well-formed pseudowords. As both variants are neologisms, the overall novelty effect may have dominated any difference between well- and ill-formed forms. In the experiment here, the second session provided an opportunity to measure this effect where the overall novelty effect was reduced by repeated presentation over a few days, or equivalently, a learning effect. In the interaction between formedness and session in the early time window, we observed that the novelty effect was most reduced for well-formed clusters, or equivalently, that well-formed clusters are easier to learn.

Similarly, the lack of an effect for existence in the N400 time window may be explained by dominance of the novelty effect across the entire word—while the clusters were individually existent, the words as a whole were not. In terms of learning, the effect for existence may have also been dominated by the learning effect for formedness.

We interpret the time window between 700 and 1050 ms to represent the late positive component due to the experimental design. In this time window, the pattern of results is somewhat more complex with an increased positivity for formedness and existence in the second session compared to the first session. In other words, both formedness and existence exhibited an effect of learning. Additionally, formedness and existence interact with each other.

Friedman and Johnson (2000) report on a range of studies in which intentional or incidental encoding was reflected by an LPC. In the present experiment, learning was incidental, as the participants' task involved intentional memorization and recollection of word-picture pairs, but did not require active attention toward the phonotactic properties of the stimuli.

In line with more recent literature, we can also consider an alternative yet broadly compatible explanation. As late positivities are often related to task and attention (Sassenhagen et al., 2014), we may view the adaption and re-orienting of attention toward previously unknown stimuli (Verleger, 1988; Sassenhagen and Bornkessel-Schlesewsky, 2015). In other words, the extra resources allocated toward successfully recognizing and processing ill-formed non-existent clusters is reflected in an increased positivity for these clusters, while the well-formed existent clusters require no additional effort and thus do not elicit a positivity.

Since formedness causes a main effect for the later time-window, while existence is significant only in interactions, we conclude that the former, instantiating the phonotactic principle of sonority, has a more dominant and immediate contribution to language processing. Generally, the lack of an effect for existence in the N400 time window is in accordance with the fact that Polish speakers have been exposed to a great array of clusters of various length and complexity (see Section Phonotactics). For example, on the basis of an exhaustive list of word-initial clusters, Orzechowska and Wiese (2015) report on 56 initial clusters in German and 423 in Polish. For final clusters, the number of clusters is still large, but comparable, in the two languages; 155 and 151 were reported for Polish and German monosyllables, respectively. Contrary to expectations (hypotheses 4 and 5), the present study demonstrated a robust learning effect for formedness, but not for existence. In other words, Polish speakers are sensitive to sonority, even though in general quite a few final clusters (almost 40%) violate the sonority restrictions (Orzechowska, 2009). Thus, we conclude that sonority constitutes a principle which is relevant even in the absence of clear positive evidence in the input patterns. It remains to be tested how this result would carry over to more complex clusters of length greater than two.

We assume, following (Nespor et al., 2003), that consonants and thereby consonant clusters play a crucial role in the creation of lexical entries and in lexical access. Furthermore, the word-final position is assumed to play a less salient role than the initial position. This is likely to result in word-final clusters' misperception, reduction and ensuing increased difficulty in their mastery. Psycholinguistic models (such as the cohort model by Marslen-Wilson, 1987) have emphasized the asymmetry between word-initial and word-final information. These facts make final clusters a more challenging subject matter for the testing of phonological processing and learnability.

Existent clusters are, by their very nature, more deeply entrenched into the mental lexicon than non-existent ones. This causes an effect of existence in the learning process, but limits its occurrence to a relatively late time-window. This is also in line with the theory by Friston (2005) of cortical responses. Sonority as a phonetic feature is closer to perception and thus processed lower on the cortical hierarchy, which is reflected in an earlier, dominant effect, compared to existence as a concept related to the over-all phonological system of the language.

Ulbrich et al. (2016) obtained similar results in an experiment of the same design but for speakers of German, a language in which consonant clusters tend to follow sonority restrictions. For these speakers, learning of a set of final clusters which were nearly identical to the set used here was facilitated if clusters adhered to the sonority principle, and if they existed in the German language. The present results on Polish are thus not confined to speakers of a specific language.

The present study provided evidence for an active role of sonority preferences in the processing of words, see also Moreton (2002) who provides evidence for the role of other structural accounts in the misperception of English consonant clusters). In a similar vein, Berent and Lennertz (2010) and Berent et al. (2014) argue that sonority restrictions indeed exemplify language universals. In an fMRI experiment with speakers of English, monosyllabic items violating these restrictions engaged (the posterior part of) Broca's area the more the items diverged from the preferred sonority profile, while the anterior part of Broca's area showed a decrease in correspondence with the preferred sonority profile. While these observations are based on the brain localization of sonority effects, the results in the present study address questions of time-related processing steps in the brain's activity. Berent et al. (2014) found evidence that *non-existent* consonant clusters in English show gradient neural responses depending on the degree of the (non-)obedience to sonority principles. Complementing these findings, our results demonstrate that even *existent* clusters lead to different responses depending on their well-formedness.

As for the debate between principle-based and usage-based phonological theories, we conclude in pointing out that there is no a priori logical reason to assume that one of the two perspectives must be correct to the extent of excluding the other. Our results (similar to those by Boll-Avetisyan and Kager, 2016) point to a scenario in which both the phonotactic principle of sonority as well as frequency-based input patterns constrain the way in which the brain of adult language-users processes and learns the complexities of language.

## Ethics statement

This study was carried out in accordance with the recommendations of *FAQ: Informationen für Geistes- und Sozialwissenschaftler/innen, Deutsche Forschungsgemeinschaft* (http://www.dfg.de/foerderung/faq/geistes_sozialwissenschaften/index.html) with written informed consent from all subjects. The *Ethikkommission der DGfS* (https://dgfs.de/de/inhalt/ueber/ethikkomission.html) approves these recommendations. All subjects gave written informed consent in accordance with the Declaration of Helsinki.

## Author contributions

All authors contributed to the planning of the study, of the analysis and interpretation of the data, and of the conception of the manuscript.

## Funding

Funding for the study reported in this paper was provided by the LOEWE initiative of the state of Hesse, Germany (Fundierung linguistischer Basiskategorien). The sponsor was not involved in any aspect of the study and/or the article.

### Conflict of interest statement

The authors declare that the research was conducted in the absence of any commercial or financial relationships that could be construed as a potential conflict of interest. The reviewer RC declared a past collaboration with one of the authors RW to the handling Editor, who ensured that the process met the standards of a fair and objective review.

## Footnotes

^1^The sonority violation in the coda is a non-monotonic decrease from the peak of the vocalic nucleus, and thus the peak of the vocalic nucleus is in some sense the start of the (critical portion of the) stimulus of interest.

^2^As in the present experiment, the response-evaluation phase in the German experiment was chosen as the relevant phase for analysis. Nonetheless, we still expect reduced effects in the stimulus-presentation phase and thus can use that phase as a conservative and non-circular estimate of the best time windows for our analysis (Kilner, 2013).

## References

[B1] Baayen R. H. Davidson D. J. Bates D. M. (2008). Mixed-effects modeling with crossed random effects for subjects and items. J. Mem. Lang. 59, 390–412. 10.1016/j.jml.2007.12.005

[B2] Bahlmann J. Schubotz R. I. Friederici A. D. (2008). Hierarchical artificial grammar processing engages Broca's area. NeuroImage 42, 525–534. 10.1016/j.neuroimage.2008.04.249 18554927

[B3] Bates D. Maechler M. Bolker B. Walker S. (2015). Fitting linear mixed-effects models using lme4. J. Stat. Softw. 67, 1–48. 10.18637/jss.v067.i01

[B4] Bentin S. Mouchetant-Rostaing Y. Giard M. H. Echallier J. F. Pernier J. (1999). ERP manifestations of processing printed words at different psycholinguistic levels: time course and scalp distribution. J. Cogn. Neurosci. 11, 235–260. 10.1162/089892999563373 10402254

[B5] Berent I. Lennertz T. (2010). Universal constraints on the sound structure of language: phonological or acoustic? J. Exp. Psychol. Hum. Percept. Perform. 36, 212–223. 10.1037/a0017638 20121305

[B6] Berent I. Lennertz T. Jun J. Moreno M. A. Smolensky P. (2008). Language universals in human brains. PNAS 105, 5321–5325. 10.1073/pnas.0801469105 18391198 PMC2291138

[B7] Berent I. Pan H. Zhao X. Epstein J. Bennett M. L. Deshpande V. Stern E.. (2014). Language universals engage Broca's area. PLoS ONE 9:e95155. 10.1371/journal.pone.0095155 24743423 PMC3990587

[B8] Boll-Avetisyan N. Kager R. (2016). Is speech processing influenced by abstract or detailed phonotactic representations? The case of the obligatory contour principle. Lingua 171, 74–91. 10.1016/j.lingua.2015.11.008

[B9] Bybee J. L. (2001). Phonology and Language Use. Cambridge: Cambridge University Press.

[B10] Bybee J. L. (2006). From usage to grammar: the mind's response to repetition. Language 82, 711–733. 10.1353/lan.2006.0186

[B11] Chambers K. E. Onishi K. H. Fisher C. (2003). Infants learn phonotactic regularities from brief auditory experience. Cognition 87, B69–B77. 10.1016/s0010-0277(02)00233-0 12590043

[B12] Chomsky N. A. Halle M. (1968). The Sound Pattern of English. New York, NY: Harper and Row.

[B13] Clements G. N. (1990). The role of the sonority cycle in core syllabification, in Papers in Laboratory Phonology I: Between the Grammar and Physics of Speech, Vol. 1, eds Kingston J. Beckman M. E. (New York, NY: Cambridge University Press), 283–333.

[B14] Clements G. N. Keyser S. J. (1983). CV Phonology. A Generative Theory of the Syllable. Cambridge, MA: Linguistic Inquiry Monographs.

[B15] Dehaene-Lambertz G. Dupoux E. Gout A. (2000). Electrophysiological correlates of phonological processing: a cross-linguistic study. J. Cogn. Neurosci. 12, 635–647. 10.1162/089892900562390 10936916

[B16] de Jong N. Wempe T. (2009). PRAAT script to detect syllable nuclei and measure speech rate automatically. Behav. Res. Methods 41, 385–390. 10.3758/BRM.41.2.385 19363178

[B17] Domahs U. Kehrein W. Knaus J. Wiese R. Schlesewsky M. (2009). Event-related potentials reflecting the processing of phonological constraint violations. Lang. Speech 52, 415–435. 10.1177/0023830909336581 20121040

[B18] Dupoux E. Kakehi K. Hirose Y. Pallier C. Mehler J. (1999). Epenthetic vowels in Japanese: a perceptual illusion? J. Exp. Psychol. Hum. Percept. Perform. 25, 1568–1578. 10.1037/0096-1523.25.6.1568

[B19] Dupoux E. Pallier C. Kakehi K. Mehler J. (2001). New evidence for prelexical phonological processing in word recognition. Lang. Cogn. Process 16, 491–505. 10.1080/01690960143000191

[B20] Dziubalska-Kołaczyk K. (1995). Phonology without the Syllable: A Study in the Natural Framework. Poznań: Motivex.

[B21] Friederici A. D. Bahlmann J. Heim S. Schubotz R. I. Anwander A. (2006). The brain differentiates human and non-human grammars: functional localization and structural connectivity. PNAS 103, 2458–2463. 10.1073/pnas.0509389103 16461904 PMC1413709

[B22] Friedman D. Johnson R. (2000). Event-Related Potential (ERP) studies of memory encoding and retrieval: a selective review. Microsc. Res. Tech. 51, 6–28. 10.1002/1097-0029(20001001)51:1<6::AID-JEMT2>3.0.CO;2-R 11002349

[B23] Friston K. (2005). A theory of cortical responses. Philos. Trans. R. Soc. Lond. B Biol. Sci. 360, 815–836. 10.1098/rstb.2005.1622 15937014 PMC1569488

[B24] Gahl S. Garnsey S. M. (2004). Knowledge of grammar, knowledge of usage: syntactic probabilities affect pronunciation variation. Language 80, 748–775. 10.1353/lan.2004.0185

[B25] Goldinger S. (1996). Words and voices: episodic traces in spoken word identification and recognition memory. J. Exp. Psychol. 22, 1166–1183. 10.1037/0278-7393.22.5.1166 8926483

[B26] Gomez R. L. Gerken L. (1999). Artificial grammar learning by 1-year-olds leads to specific and abstract knowledge. Cognition 70, 109–135. 10.1016/S0010-0277(99)00003-7 10349760

[B27] Gómez R. L. Gerken L. (2000). Infant artificial language learning and language acquisition. Trends Cogn. Sci. 4, 178–186. 10.1016/S1364-6613(00)01467-4 10782103

[B28] Greenberg J. H. (1978). Some generalizations concerning initial and final consonant clusters, in Universals of Human Language Vol. 2, ed Greenberg J. H. (Stanford, CA: Stanford University Press), 243–279.

[B29] Hart J. 't. Collier R. Cohen A. (1990). A Perceptual Study of Intonation. An Experimental-phonetic Approach to Speech Melody. Cambridge: Cambridge University Press.

[B30] Hoole P. Bombien L. Pouplier M. Mooshammer C. Kühnert B. (Eds.). (2012). Consonant Clusters and Structural Complexity. Berlin/Boston, MA: Walter de Gruyter.

[B31] Hooper J. B. (1976). An Introduction to Natural Generative Phonology. New York, NY: Academic Press.

[B32] International Phonetic Association (Ed.). (2007). Handbook of the International Phonetic Association: A Guide to the Use of the International Phonetic Alphabet, 9th Edn. Cambridge: Cambridge University Press.

[B33] Jassem W. (2003). Polish. J. Int. Phon. Assoc. 33, 103–107. 10.1017/S002510030300119127279822

[B34] Jusczyk P. W. Luce P. A. Charles-Luce J. (1994). Infants' sensitivity to phonotactic patterns in the native language. J. Mem. Lang. 33, 630–645. 10.1006/jmla.1994.1030

[B35] Kabak B. Idsardi W. J. (2007). Perceptual distortions in the adaptation of English consonant clusters: syllable structure or consonantal contact constraints? Lang. Speech 50, 23–52. 10.1177/00238309070500010201 17518102

[B36] Kilner J. M. (2013). Bias in a common EEG and MEG statistical analysis and how to avoid it. Clin. Neurophysiol. 124, 2062–2063. 10.1016/j.clinph.2013.03.024 23639379

[B37] Kutas M. Federmeier K. D. (2011). Thirty years and counting: finding meaning in the N400 component of the event-related brain potential (ERP). Annu.Rev. Psychol. 62, 621–647. 10.1146/annurev.psych.093008.131123 20809790 PMC4052444

[B38] Laver J. (1994). Principles of Phonetics. Cambridge: Cambridge University Press.

[B39] Maess B. Schröger E. Widmann A. (2016). High-pass filters and baseline correction in M/EEG analysis-continued discussion. J. Neurosci. Methods 266, 171–172. 10.1016/j.jneumeth.2016.01.016 26812439

[B40] Marslen-Wilson W. (1987). Functional parallelism in spoken word recognition. Cognition 25, 71–102. 10.1016/0010-0277(87)90005-9 3581730

[B41] McCandliss B. D. Posner M. I. Givón T. (1997). Brain plasticity in learning visual words. Cogn. Psychol. 33, 88–110. 10.1006/cogp.1997.0661

[B42] Meinhold G. Stock E. (1980). Phonologie der deutschen Gegenwartssprache. Leipzig: VEB Bibliographisches Institut Leipzig.

[B43] Moore-Cantwell C. Pater J. Staubs R. Zobel B. Sanders L. (2013). ERP Indices of Lab-Learned Phonotactics. Amherst, MA: Presented at the RUMMIT.

[B44] Moreton E. (2002). Structural constraints in the perception of English stop-sonorant clusters. Cognition 84, 55–71. 10.1016/S0010-0277(02)00014-8 12062147

[B45] Mueller J. L. Hahne A. Fujii Y. Friederici A. D. (2005). Native and non-native speakers' processing of a miniature version in Japanese as revealed by ERPs. J. Cogn. Neurosci. 17, 1229–1244. 10.1162/0898929055002463 16197680

[B46] Munson B. (2001). Phonological pattern frequency and speech production in adults and children. J. Speech Lang. Hear. Res. 44, 778–792. 10.1044/1092-4388(2001/061) 11521771

[B47] Nespor M. Peña M. Mehler J. (2003). On the different roles of vowels and consonants in speech processing and language acquisition. Lingue Linguaggio 2, 203–230. 10.1418/10879

[B48] Nooteboom S. G. (1997). The prosody of speech: melody and rhythm, in The Handbook of Phonetic Sciences, eds Hardcastle W. Laver J. (Oxford: Blackwell), 640–673.

[B49] Onishi K. H. Chambers K. E. Fisher C. (2002). Learning phonotactic constraints from brief auditory experience. Cognition 83, B13–B23. 10.1016/S0010-0277(01)00165-2 11814489

[B50] Orzechowska P. (2009). Morphonotactics in English and Polish. A Dictionary- and Corpus-Based Study of Word-Final Consonant Clusters. Ph. D. dissertation, Adam Mickiewicz University, Poznań.

[B51] Orzechowska P. (2016). In search of phonotactic preferences, in Yearbook Poznan Linguistic Meeting, Vol. 2, 167–193. 10.1515/yplm-2016-0008

[B52] Orzechowska P. Wiese R. (2015). Preferences and variation in phonotactics: a multi-dimensional evaluation of German and Polish. Folia Linguist. 49, 439–486. 10.1515/flin-2015-0016

[B53] Parker S. (Ed.). (2012). The Sonority Controversy. Berlin/Boston, MA: Walter de Gruyter.

[B54] Pierrehumbert J. (2001). Exemplar dynamics: Word frequency, lenition and contrast, in Frequency and the Emergence of Linguistic Structure, eds Bybee J. L. Hoppper P. (Philadelphia: John Benjamins), 137–158.

[B55] Prince A. S. Smolensky P. (1993). Optimality Theory. Constraint Interaction in Generative Grammar. Malden, MA; Oxford: Blackwell.

[B56] Pukui M. K. Elbert S. H. (1979). Hawaiian Grammar. Honolulu: University of Hawaii Press.

[B57] Redford M. A. (2008). Production constraints on learning novel onset phonotactics. Cognition 107, 785–816. 10.1016/j.cognition.2007.11.014 18237726

[B58] Rice K. D. (1992). On deriving sonority: a structural account of sonority relationships. Phonology 9, 61–99. 10.1017/S0952675700001500

[B59] Rietveld A. C. M. Gussenhoven C. (1985). On the relation between pitch excursion size and prominence. J. Phon. 13, 299–308.

[B60] Romani C. Galluzzi C. (2005). Effects of syllabic complexity in predicting accuracy of repetition and direction of errors in patients with articulatory and phonological difficulties. Cogn. Neuropsychol. 22, 817–850. 10.1080/02643290442000365 21038278

[B61] Rossi S. Jürgenson I. B. Hanulíkova A. Telkemeyer S. Wartenburger I. Obrig H. (2011). Implicit processing of phonotactic cues: evidence from electrophysiological and vascular responses. J. Cogn. Neurosci. 23, 1752–1764. 10.1162/jocn.2010.21547 20666594

[B62] Rubach J. (1996). Nonsyllabic analysis of voice assimilation in polish. Linguist. Inq. 27, 69–110.

[B63] Rubach J. Booij G. E. (1990). Edge of constituent effects in Polish. Nat. Lang. Linguist. Theory 8, 427–463. 10.1007/BF00135620

[B64] Sassenhagen J. Alday P. M. (2016). A common misapplication of statistical inference: nuisance control with null-hypothesis significance tests. Brain Lang. 162, 42–45. 10.1016/j.bandl.2016.08.001 27543688

[B65] Sassenhagen J. Bornkessel-Schlesewsky I. (2015). The P600 as a correlate of ventral attention network reorientation. Cortex 66, A3–A20. 10.1016/j.cortex.2014.12.019 25791606

[B66] Sassenhagen J. Schlesewsky M. Bornkessel-Schlesewsky I. (2014). The P600-as-P3 hypothesis revisited: single-trial analyses reveal that the late EEG positivity following linguistically deviant material is reaction time aligned. Brain Lang. 137, 29–39. 10.1016/j.bandl.2014.07.010 25151545

[B67] Seiler H. (1962). Laut und Sinn: zur Struktur der deutschen Einsilbler. Lingua 11, 375–387. 10.1016/0024-3841(62)90047-5

[B68] Selkirk E. O. (1984). On the major class features and syllable theory, in Language Sound Structure, eds Mark A. Oehrle R. T. (Cambridge MA: The MIT Press), 107–136.

[B69] Sievers E. (1901). Grundzüge der Phonetik. Zur Einführung in das Studium der Lautlehre der indogermanischen Sprachen. Leipzig: Breitkopf & Härtel.

[B70] Sommerstein A. H. (1974). On phonotactically motivated rules. J. Linguist. 10, 71–94. 10.1017/S0022226700004011

[B71] Steriade D. (1999). Alternatives to the syllabic interpretation of consonantal phonotactics, in Item Order in Language and Speech, eds Fujimura O. Joseph B. D. Palek B. (Columbus, OH: The Ohio State University; The Karolinum Press), 205–242.

[B72] Treiman R. Fowler C. A. Gross J. Berch D. Weatherston S. (1995). Syllable structure or word structure? Evidence for onset and rime units with disyllabic and trisyllabic stimuli. J. Mem. Lang. 34, 132–155. 10.1006/jmla.1995.10072072673

[B73] Trubetzkoy N. S. (1967). Grundzüge der Phonologie, 2nd Edn. Göttingen: Vandenhoeck & Ruprecht.

[B74] Ulbrich C. Alday P. Knaus J. Orzechowska P. Wiese R. (2016). The role of phonotactic principles in language processing. Lang. Cogn. Neurosci. 31, 662–682. 10.1080/23273798.2015.1136427

[B75] Verleger R. (1988). Event-related potentials and cognition: a critique of the context updating hypothesis and an alternative interpretation of P3. Behav. Brain Sci. 11, 343–356. 10.1017/S0140525X00058015

[B76] Vitevitch M. S. Luce P. A. Charles-Luce J. Kemmerer D. (1997). Phonotactics and syllable stress: implications for the processing of spoken nonsense words. Lang. Speech 40, 47–62. 9230698 10.1177/002383099704000103

[B77] Westbury C. Buchanan L. Brown N. R. (2002). Sounds of the neighborhood: false memories and the structure of the phonological lexicon. J. Mem. Lang. 46, 622–651. 10.1006/jmla.2001.2821

[B78] Whitney W. D. (1865). On the relation of vowels and consonants. J. Am. Orient. Soc. 8, 357–373.

[B79] Wright C. E. (1979). Duration differences between rare and common words and their implications for the interpretation of word frequency effects. Mem. Cogn. 7, 411–419. 10.3758/BF03198257 542114

[B80] Zydorowicz P. Orzechowska P. Jankowski M. Dziubalska-Kołaczyk K. Wierzchoń P. Pietrala D. (2016). Phonotactics and morphonotactics of Polish and English. Theory, Description, Tools and Applications. Poznań: Wydawnictwo Naukowe UAM.

