# Supplementary material for: Structural Principles or Frequency of Use? An ERP Experiment on the Learnability of Consonant Clusters
Source: Front Psychol. 2017 Jan 9;7:2005. doi: 10.3389/fpsyg.2016.02005 (PMC5220188; doi:10.3389/fpsyg.2016.02005)
Supplement: Supplementary file 1 [file Table1.pdf]

Appendix 1

Final clusters and Polish example word

Existent (EX): Well-formed (WF)	Existent (EX): Ill-formed (IF)
ɕp – próśb 'request' (Gen.pl) > prośb+a<br>ɕtɕ – kość 'bone'<br>fk – tomahawk (axe)<br>ftɕ – sprawdź! 'check' (Voc. sg.) > sprawdz+ić<br>jm – sejm 'parliament'<br>jp – knajp 'tavern' (Gen. pl) > knajp+a<br>js – rejs 'race'<br>lk – walk 'fight' (Gen. pl) > walk+a<br>lm – palm 'palm tree' (Gen. pl) > palm+a<br>mɕ – kimś 'somebody's'<br>mʃ – zamsz 'suede'<br>mx – czeremch 'hackberry' (Gen. pl) > czeremch+a<br>ɲtɕ – chęć 'willingness'<br>rk – cyrk 'circus'<br>rs – kurs 'course'<br>sk – kask 'helmet'<br>sp – zasp 'snowdrift' (Gen. pl) > zasp+a<br>ʃt – koszt 'cost'<br>ʃʧ – bluszcz 'ivy'<br>ʧp – liczb 'number' (Gen. pl) > liczb+a<br>ʧt – uczt 'celebration' (Gen. pl) > uczt+a	ɕl – myśl 'thought'<br>fn – hafn 'hafnium'<br>fr – szyfr 'cipher'<br>kf – strzykw 'sea cucumber (Gen. pl) > strzykw+a<br>kl – cykl 'cycle'<br>km – flegm 'flegm' (Gen. pl) > flegm+a<br>kx – sikh 'sikh'<br>mn – hymn 'anthem'<br>nr – henr 'henry'<br>pɲ – wapń 'calcium'<br>ps – gips 'plaster'<br>pt – szept 'whisper'<br>ptɕ – gapć 'butterfingers' (Gen. pl) > gapci+a<br>pʧ – depcz! 'tread' (Voc. sg)<br>ʃx – zmierzch 'twillight'<br>tf – tratw 'raft' (Gen. pl) > tratw+a<br>tr – wiatr 'wind'<br>xm – drachm 'drachma' (Gen. pl) > drachm+a<br>xʃ – spichrz 'breadbasket'
