# Supplementary material for: Structural Principles or Frequency of Use? An ERP Experiment on the Learnability of Consonant Clusters
Source: Front Psychol. 2017 Jan 9;7:2005. doi: 10.3389/fpsyg.2016.02005 (PMC5220188; doi:10.3389/fpsyg.2016.02005)
Supplement: Supplementary file 2 [file Table2.pdf]

Appendix 2

Generalized linear mixed model fit by maximum likelihood (Laplace Approximation) ['glmerMod']
Family: binomial (logit)
Formula: resp ~ session * existence * formedness + (1 + existence + formedness | subj) + (1 | item)
Control: glmerControl(optimizer = "bobyqa", optCtrl = list(maxfun = 1e+07))

AIC	BIC	logLik	deviance	df.resid
12569.9	12678.8	-6270.0	12539.9	10464

Scaled residuals:

Min	1Q	Median	3Q	Max
-2.8837	-1.1156	0.5348	0.6933	1.3054

Random effects:

Groups	Name	Variance	Std.Dev.	Corr	
item	(Intercept)	0.042445	0.20602		
subj	(Intercept)	0.147992	0.38470		
	existence[existent]	0.001964	0.04431	1.00	
	formedness[well]	0.001361	0.03689	-1.00	-1.00

Number of obs: 10479, groups: item, 63; subj, 22

Fixed effects:

	Estimate	Std. Error	z	Pr(>|z|)	
(Intercept)	0.821367	0.088816	9.248	<2e-16	***
session[1]	-0.309370	0.021862	-14.151	<2e-16	***
existence[existent]	0.035437	0.023879	1.484	0.1378	
formedness[well]	0.039165	0.023286	1.682	0.0926	.
session[1]:existence[existent]	-0.021314	0.021775	-0.979	0.3276	
session[1]:formedness[well]	-0.042632	0.021774	-1.958	0.0502	.
existence[existent]:formedness[well]	0.016710	0.021750	0.768	0.4423	
session[1]:existence[existent]:formedness[well]	-0.001693	0.021745	-0.078	0.9380	

Significance Codes: '***' 0.001, '.' 0.1

Correlation of Fixed Effects:

	(Intr)	sss[1]	exst[]	frmd[]	sssn[1]:x[]	sssn[1]:f[]	e[]:[]
session[1]	-0.021						
exstnc[xst]	0.371	-0.013					
frmdnss[wl]	-0.308	-0.020	-0.128				
sssn[1]:x[]	-0.004	0.022	-0.076	-0.007			
sssn[1]:f[]	-0.006	0.016	-0.007	-0.078	0.006		
exstnc[]:[]	0.002	-0.008	0.014	0.021	-0.022	-0.013	
ss[1]:[]:[]	-0.002	0.006	-0.020	-0.012	0.017	0.020	-0.077
