# Supplementary material for: Structural Principles or Frequency of Use? An ERP Experiment on the Learnability of Consonant Clusters
Source: Front Psychol. 2017 Jan 9;7:2005. doi: 10.3389/fpsyg.2016.02005 (PMC5220188; doi:10.3389/fpsyg.2016.02005)
Supplement: Supplementary file 3 [file Table3.pdf]

Appendix 3

ANOVA-type output restricted to significant effects; Analysis of Deviance Table (Type II Wald chisquare tests)
Formula: mean ~ session * roi * existence * formedness * F0 * duration * intensity + (1 + session + existence + formedness | subj) + (1 + session + existence + formedness | item)

(1) Time-window 450-550ms.
Response: mean

	Chisq	Df	Pr(>Chisq)	
session	11.1519	1	0.0008394	***
roi	419.6473	1	<2.2e-16	***
int	7.3660	1	0.0066467	**
session:roi	53.1963	1	3.018e-13	***
session:formedness	6.5098	1	0.0107281	*
session:F0	8.4695	1	0.0036116	**
existence:F0	13.6261	1	0.0002231	***
roi:int	14.6370	1	0.0001303	***
existence:int	11.8106	1	0.0005889	***
F0:int	31.9588	1	1.575e-08	***
dur:int	7.9346	1	0.0048497	**
existence:formedness:F0	8.3315	1	0.0038963	**
session:existence:dur	10.0362	1	0.0015349	**
existence:formedness:dur	4.8335	1	0.0279125	*
existence:F0:dur	7.8586	1	0.0050578	**
formedness:F0:dur	12.5848	1	0.0003889	***
session:existence:int	10.5120	1	0.0011860	**
session:formedness:int	18.0078	1	2.200e-05	***
session:F0:int	7.7167	1	0.0054714	**
formedness:dur:int	9.0846	1	0.0025778	**
F0:dur:int	14.8394	1	0.0001171	***
session:existence:formedness:dur	30.4638	1	3.402e-08	***
roi:existence:formedness:dur	4.3695	1	0.0365884	*
session:existence:F0:dur	5.8918	1	0.0152114	*
session:existence:F0:int	13.3511	1	0.0002583	***
session:formedness:F0:int	5.9446	1	0.0147623	*
existence:formedness:F0:int	7.6709	1	0.0056120	**
session:formedness:dur:int	7.0879	1	0.0077606	**
existence:formedness:dur:int	4.6925	1	0.0302950	*
session:existence:formedness:dur:int	4.4575	1	0.0347489	*
session:formedness:F0:dur:int	6.1976	1	0.0127923	*
existence:formedness:F0:dur:int	13.4911	1	0.0002397	***

Significance Codes: '***' 0.001, '**' 0.01, '*' 0.05

(2) Time-window 750-1050ms.
Response: mean

	Chisq	Df	Pr(>Chisq)	
session	5.3865	1	5.3865	*
roi	724.8985	1	724.8985	***
formedness	5.2410	1	5.2410	*
F0	25.2832	1	25.2832	***
session:roi	21.8537	1	21.8537	***
session:existence	5.1535	1	5.1535	*
session:formedness	5.3824	1	5.3824	*
existence:formedness	9.2795	1	9.2795	**
roi:F0	6.2408	1	6.2408	*
existence:F0	4.4374	1	4.4374	*
roi:dur	9.9931	1	9.9931	**
F0:dur	10.9094	1	10.9094	***
formedness:int	21.0450	1	21.0450	***
F0:int	18.7936	1	18.7936	***
dur:int	16.1325	1	16.1325	***
roi:existence:formedness	5.4697	1	5.4697	*
existence:formedness:F0	9.0874	1	9.0874	**
roi:existence:dur	10.3755	1	10.3755	**
session:formedness:dur	6.8777	1	6.8777	**
session:F0:dur	4.8166	1	4.8166	*
formedness:F0:dur	24.4786	1	24.4786	***
session:existence:int	5.9762	1	5.9762	*
existence:formedness:int	5.1768	1	5.1768	*
session:F0:int	4.4971	1	4.4971	*
formedness:dur:int	13.1199	1	13.1199	***
session:existence:formedness:F0	21.1799	1	21.1799	***
session:formedness:F0:dur	11.0860	1	11.0860	***
existence:formedness:F0:dur	5.7664	1	5.7664	*
session:roi:formedness:int	7.4510	1	7.4510	**
roi:existence:formedness:int	3.8751	1	3.8751	*
session:existence:F0:int	7.2937	1	7.2937	**
existence:F0:dur:int	6.5239	1	6.5239	*
session:existence:formedness:dur:int	4.8462		4.8462	*
session:existence:F0:dur:int	8.9143		8.9143	**

Significance Codes: '***' 0.001, '**' 0.01, '*' 0.05
