# Supplementary material for: Structural Principles or Frequency of Use? An ERP Experiment on the Learnability of Consonant Clusters
Source: Front Psychol. 2017 Jan 9;7:2005. doi: 10.3389/fpsyg.2016.02005 (PMC5220188; doi:10.3389/fpsyg.2016.02005)
Supplement: Supplementary file 4 [file Table4.pdf]

Appendix 4

Full model summary; Linear mixed model fit by maximum likelihood ['lmerMod']
Formula: mean ~ session * roi * existence * formedness * F0 * duration * intensity + (1 + session + existence + formedness | subj) + 1 + session + existence + formedness | item
Control: lmerControl(optimizer = "nloptwrap", calc.derivs = TRUE)

(1) Time-window 450-550 ms.

AIC	BIC	logLik	deviance	df.resid
721391.0	722820.6	360546.5	721093.0	108351

Scaled residuals:

Min	1Q	Median	3Q	Max
-11.1590	0.594	0.0088	0.6117	11.6469

Random effects:

Groups	Name	Variance	Std.Dev.	Corr		
item	(Intercept)	0.4197	0.6479			
	session1	0.8200	0.9055	0.03		
	existence1	1.2312	1.1096	0.32	-0.18	
	formedness1	1.4092	1.1871	0.08	0.09	0.24
subj	(Intercept)	4.6306	2.1519			
	session1	2.2162	1.4887	-0.22		
	existence1	0.2201	0.4691	-0.01	0.39	
	formedness1	0.3695	0.6079	0.52	-0.24	-0.27
Residual		44.6742	6.6839			

Number of obs: 108500, groups: item, 63; subj, 23

	Estimate	Std. Error	t value
(Intercept)	-3.220987	0.462923	-6.958
ses.2	0.858995	0.343335	2.502
post.	0.872227	0.081653	10.682
non.exist	0.717680	0.225582	3.181
wellform.	0.156655	0.258711	0.606
F0	-0.486177	0.132847	-3.660
dur	-0.364755	0.088246	-4.133
int	0.338849	0.078040	4.342
ses.2:post.	-0.578794	0.163306	-3.544
ses.2:non.exist	0.323414	0.177960	1.817
post.:non.exist	-0.056074	0.163306	-0.343
ses.2:wellform.	1.061920	0.186959	5.680
post.:wellform.	-0.088376	0.163306	-0.541
non.exist:wellform.	-0.346465	0.301787	-1.148
ses.2:F0	-0.059064	0.140073	-0.422
post.:F0	0.116712	0.114836	1.016

non.exist:F0	0.504690	0.252316	2.000
wellform.:F0	-0.168151	0.251204	-0.669
ses.2:dur	-0.499377	0.106402	-4.693
post.:dur	-0.034490	0.094760	-0.364
non.exist:dur	-0.109266	0.194245	-0.563
wellform.:dur	0.179036	0.197968	0.904
F0:dur	-0.285119	0.135947	-2.097
ses.2:int	0.159065	0.092921	1.712
post.:int	-0.193231	0.082721	-2.336
non.exist:int	0.668530	0.165676	4.035
wellform.:int	0.053019	0.169347	0.313
F0:int	0.282440	0.123208	2.292
dur:int	-0.097555	0.093148	-1.047
ses.2:post.:non.exist	0.435471	0.326611	1.333
ses.2:post.:wellform.	0.068307	0.326611	0.209
ses.2:non.exist:wellform.	0.202573	0.368879	0.549
post.:non.exist:wellform.	-0.226363	0.326611	-0.693
ses.2:post.:F0	0.038857	0.229672	0.169
ses.2:non.exist:F0	-0.544965	0.266577	-2.044
post.:non.exist:F0	-0.065946	0.229672	-0.287
ses.2:wellform.:F0	0.212179	0.256482	0.827
post.:wellform.:F0	-0.204284	0.229672	-0.889
non.exist:wellform.:F0	-2.101911	0.490393	-4.286
ses.2:post.:dur	-0.273540	0.189520	-1.443
ses.2:non.exist:dur	0.595200	0.215468	2.762
post.:non.exist:dur	-0.103162	0.189520	-0.544
ses.2:wellform.:dur	0.488820	0.219879	2.223
post.:wellform.:dur	-0.124192	0.189520	-0.655
non.exist:wellform.:dur	-1.613370	0.424516	-3.800
ses.2:F0:dur	0.203454	0.150702	1.350
post.:F0:dur	0.208617	0.130034	1.604
non.exist:F0:dur	1.367004	0.298921	4.573
wellform.:F0:dur	1.359277	0.272470	4.989
ses.2:post.:int	-0.078949	0.165442	-0.477
ses.2:non.exist:int	0.659210	0.187497	3.516
post.:non.exist:int	-0.182519	0.165442	-1.103
ses.2:wellform.:int	0.771083	0.190941	4.038
post.:wellform.:int	-0.203307	0.165442	-1.229
non.exist:wellform.:int	0.588720	0.340761	1.728
ses.2:F0:int	-0.303559	0.135112	-2.247
post.:F0:int	0.034318	0.119125	0.288
non.exist:F0:int	-0.603045	0.273854	-2.202

wellform.:F0:int	-1.046805	0.254067	-4.120
ses.2:dur:int	-0.189560	0.109469	-1.732
post.:dur:int	0.002456	0.094920	0.026
non.exist:dur:int	-0.458848	0.191920	-2.391
wellform.:dur:int	-0.628718	0.191323	-3.286
F0:dur:int	0.643013	0.138026	4.659
ses.2:post.:non.exist:wellform.	0.080679	0.653223	0.124
ses.2:post.:non.exist:F0	-0.090896	0.459344	-0.198
ses.2:post.:wellform.:F0	-0.098080	0.459344	-0.214
ses.2:non.exist:wellform.:F0	0.431805	0.540775	0.798
post.:non.exist:wellform.:F0	0.156582	0.459344	0.341
ses.2:post.:non.exist:dur	0.061694	0.379040	0.163
ses.2:post.:wellform.:dur	-0.363272	0.379040	-0.958
ses.2:non.exist:wellform.:dur	-2.507032	0.454635	-5.514
post.:non.exist:wellform.:dur	0.742714	0.379040	1.959
ses.2:post.:F0:dur	0.004913	0.260069	0.019
ses.2:non.exist:F0:dur	0.767662	0.312159	2.459
post.:non.exist:F0:dur	-0.052995	0.260069	-0.204
ses.2:wellform.:F0:dur	0.376761	0.297108	1.268
post.:wellform.:F0:dur	-0.120276	0.260069	-0.462
non.exist:wellform.:F0:dur	-0.397251	0.510711	-0.778
ses.2:post.:non.exist:int	0.082383	0.330885	0.249
ses.2:post.:wellform.:int	-0.236325	0.330885	-0.714
ses.2:non.exist:wellform.:int	0.141326	0.396805	0.356
post.:non.exist:wellform.:int	-0.452001	0.330885	-1.366
ses.2:post.:F0:int	-0.093006	0.238250	-0.390
ses.2:non.exist:F0:int	0.399745	0.288943	1.383
post.:non.exist:F0:int	0.044937	0.238250	0.189
ses.2:wellform.:F0:int	-0.568104	0.274454	-2.070
post.:wellform.:F0:int	-0.140250	0.238250	-0.589
non.exist:wellform.:F0:int	-1.400148	0.492854	-2.841
ses.2:post.:dur:int	0.056161	0.189839	0.296
ses.2:non.exist:dur:int	-0.117845	0.214172	-0.550
post.:non.exist:dur:int	0.049762	0.189839	0.262
ses.2:wellform.:dur:int	-0.563255	0.221933	-2.538
post.:wellform.:dur:int	0.117731	0.189839	0.620
non.exist:wellform.:dur:int	-0.893312	0.373941	-2.389
ses.2:F0:dur:int	0.103639	0.154129	0.672
post.:F0:dur:int	0.113680	0.125569	0.905
non.exist:F0:dur:int	0.273067	0.256437	1.065
wellform.:F0:dur:int	-0.036171	0.258001	-0.140
ses.2:post.:non.exist:wellform.:F0	-0.480747	0.918689	-0.523

ses.2:post.:non.exist:wellform.:dur	0.137989	0.758080	0.182
ses.2:post.:non.exist:F0:dur	0.028865	0.520138	0.055
ses.2:post.:wellform.:F0:dur	0.605620	0.520138	1.164
ses.2:non.exist:wellform.:F0:dur	0.438286	0.590839	0.742
post.:non.exist:wellform.:F0:dur	0.245473	0.520138	0.472
ses.2:post.:non.exist:wellform.:int	0.570288	0.661769	0.862
ses.2:post.:non.exist:F0:int	-0.315217	0.476500	-0.662
ses.2:post.:wellform.:F0:int	-0.021218	0.476500	-0.045
ses.2:non.exist:wellform.:F0:int	-0.861523	0.544067	-1.583
post.:non.exist:wellform.:F0:int	-0.022045	0.476500	-0.046
ses.2:post.:non.exist:dur:int	-0.073646	0.379678	-0.194
ses.2:post.:wellform.:dur:int	-0.258577	0.379678	-0.681
ses.2:non.exist:wellform.:dur:int	-0.958871	0.443167	-2.164
post.:non.exist:wellform.:dur:int	0.083158	0.379678	0.219
ses.2:post.:F0:dur:int	0.122195	0.251138	0.487
ses.2:non.exist:F0:dur:int	0.368725	0.286052	1.289
post.:non.exist:F0:dur:int	0.103107	0.251138	0.411
ses.2:wellform.:F0:dur:int	0.705165	0.282166	2.499
post.:wellform.:F0:dur:int	0.125681	0.251138	0.500
non.exist:wellform.:F0:dur:int	1.911435	0.520906	3.669
ses.2:post.:non.exist:wellform.:F0:dur	-0.330034	1.040276	-0.317
ses.2:post.:non.exist:wellform.:F0:int	0.072157	0.952999	0.076
ses.2:post.:non.exist:wellform.:dur:int	-0.151834	0.759357	-0.200
ses.2:post.:non.exist:F0:dur:int	-0.123039	0.502277	-0.245
ses.2:post.:wellform.:F0:dur:int	-0.088931	0.502277	-0.177
ses.2:non.exist:wellform.:F0:dur:int	0.666927	0.575051	1.160
post.:non.exist:wellform.:F0:dur:int	0.212085	0.502277	0.422
ses.2:post.:non.exist:wellform.:F0:dur:int	0.819308	1.004554	0.816

(2) Time-window 750-1050 ms.

AIC	BIC	logLik	deviance	df.resid
677140.1	678569.7	-338421.0	676842.1	108351

Scaled residuals:

Min	1Q	Median	3Q	Max
-12.2937	-0.6126	-0.0017	0.6150	12.4465

Random effects:

Groups	Name	Variance	Std.Dev.	Corr		
item	(Intercept)	0.2877	0.5363			
	session1	0.4415	0.6645	0.12		
	existence1	0.8684	0.9319	-0.23	-0.15	
	formedness1	0.8802	0.9382	0.18	-0.22	0.23
subj	(Intercept)	3.2981	1.8161			
	session1					
	existence1	0.2629	0.5127	0.13	-0.18	
	formedness1	0.1883	0.4339	-0.18	-0.15	0.39
Residual		29.7094	5.4506			

Number of obs: 108500, groups: item, 63; subj, 23

	Estimate	Std.Error	tvalue
(Intercept)	-2.157379	0.389933	-5.533
ses.2	0.618731	0.311130	1.989
post.	0.704448	0.066587	10.579
non.exist	0.260691	0.198990	1.310
wellform.	0.553691	0.203935	2.715
F0	-0.172106	0.108658	-1.584
dur	0.062343	0.071592	0.871
int	-0.025210	0.063544	-0.397
ses.2:post.	-0.464180	0.133174	-3.486
ses.2:non.exist	0.531190	0.144633	3.673
post.:non.exist	0.049399	0.133174	0.371
ses.2:wellform.	0.573209	0.151591	3.781
post.:wellform.	-0.002526	0.133174	-0.019
non.exist:wellform.	-1.034252	0.253284	-4.083
ses.2:F0	-0.154953	0.113230	-1.368
post.:F0	0.065475	0.093648	0.699
non.exist:F0	0.525665	0.210072	2.502
wellform.:F0	0.307490	0.208060	1.478
ses.2:dur	-0.182897	0.086395	-2.117
post.:dur	0.048781	0.077276	0.631
non.exist:dur	0.284366	0.154482	1.841

wellform.:dur	0.635882	0.165270	3.848
F0:dur	-0.226445	0.114201	-1.983
ses.2:int	0.076881	0.075453	1.019
post.:int	-0.044128	0.067458	-0.654
non.exist:int	-0.064384	0.133809	-0.481
wellform.:int	-0.584611	0.139361	-4.195
F0:int	0.349302	0.100263	3.484
dur:int	-0.136663	0.074997	-1.822
ses.2:post.:non.exist	0.088640	0.266348	0.333
ses.2:post.:wellform.	-0.381927	0.266348	-1.434
ses.2:non.exist:wellform.	-0.398069	0.299325	-1.330
post.:non.exist:wellform.	-0.635971	0.266348	-2.388
ses.2:post.:F0	-0.071115	0.187295	-0.380
ses.2:non.exist:F0	0.429906	0.216062	1.990
post.:non.exist:F0	-0.168703	0.187295	-0.901
ses.2:wellform.:F0	0.321968	0.208314	1.546
post.:wellform.:F0	0.130217	0.187295	0.695
non.exist:wellform.:F0	0.991585	0.395209	2.509
ses.2:post.:dur	-0.138689	0.154552	-0.897
ses.2:non.exist:dur	0.228555	0.174886	1.307
post.:non.exist:dur	0.453296	0.154552	2.933
ses.2:wellform.:dur	0.470116	0.178312	2.636
post.:wellform.:dur	0.066045	0.154552	0.427
non.exist:wellform.:dur	-0.654879	0.354270	-1.849
ses.2:F0:dur	-0.266192	0.122223	-2.178
post.:F0:dur	0.079263	0.106042	0.747
non.exist:F0:dur	0.104955	0.248778	0.422
wellform.:F0:dur	-0.734593	0.219058	-3.353
ses.2:post.:int	-0.005199	0.134916	-0.039
ses.2:non.exist:int	0.223360	0.152167	1.468
post.:non.exist:int	0.016658	0.134916	0.123
ses.2:wellform.:int	0.356958	0.154873	2.305
post.:wellform.:int	-0.105787	0.134916	-0.784
non.exist:wellform.:int	0.612284	0.278726	2.197
ses.2:F0:int	-0.240860	0.109677	-2.196
post.:F0:int	-0.020566	0.097145	-0.212
non.exist:F0:int	-0.111219	0.224438	-0.496
wellform.:F0:int	0.166323	0.204066	0.815
ses.2:dur:int	-0.042970	0.088771	-0.484
post.:dur:int	-0.015615	0.077406	-0.202
non.exist:dur:int	0.098150	0.156669	0.626
wellform.:dur:int	-0.586302	0.159618	-3.673

F0:dur:int	0.156047	0.115985	1.345
ses.2:post.:non.exist:wellform.	-0.374849	0.532696	-0.704
ses.2:post.:non.exist:F0	-0.091457	0.374590	-0.244
ses.2:post.:wellform.:F0	0.036405	0.374590	0.097
ses.2:non.exist:wellform.:F0	-1.622206	0.437850	-3.705
post.:non.exist:wellform.:F0	-0.524271	0.374590	-1.400
ses.2:post.:non.exist:dur	0.275969	0.309103	0.893
ses.2:post.:wellform.:dur	-0.136450	0.309103	-0.441
ses.2:non.exist:wellform.:dur	-0.576259	0.368071	-1.566
post.:non.exist:wellform.:dur	-0.052562	0.309103	-0.170
ses.2:post.:F0:dur	0.071521	0.212083	0.337
ses.2:non.exist:F0:dur	0.390245	0.252578	1.545
post.:non.exist:F0:dur	0.123004	0.212083	0.580
ses.2:wellform.:F0:dur	0.637641	0.240944	2.646
post.:wellform.:F0:dur	0.323244	0.212083	1.524
non.exist:wellform.:F0:dur	-0.989188	0.414485	-2.387
ses.2:post.:non.exist:int	-0.192826	0.269833	-0.715
ses.2:post.:wellform.:int	-0.572236	0.269833	-2.121
ses.2:non.exist:wellform.:int	0.172899	0.321184	0.538
post.:non.exist:wellform.:int	-0.404162	0.269833	-1.498
ses.2:post.:F0:int	-0.106899	0.194290	-0.550
ses.2:non.exist:F0:int	0.802234	0.233645	3.434
post.:non.exist:F0:int	-0.026377	0.194290	-0.136
ses.2:wellform.:F0:int	0.053224	0.222458	0.239
post.:wellform.:F0:int	-0.023626	0.194290	-0.122
non.exist:wellform.:F0:int	0.348588	0.399632	0.872
ses.2:post.:dur:int	0.210361	0.154812	1.359
ses.2:non.exist:dur:int	0.161407	0.173893	0.928
post.:non.exist:dur:int	0.002291	0.154812	0.015
ses.2:wellform.:dur:int	0.258916	0.179797	1.440
post.:wellform.:dur:int	0.004894	0.154812	0.032
non.exist:wellform.:dur:int	0.299790	0.311669	0.962
ses.2:F0:dur:int	-0.174092	0.124550	-1.398
post.:F0:dur:int	0.118950	0.102400	1.162
non.exist:F0:dur:int	-0.538816	0.215459	-2.501
wellform.:F0:dur:int	0.311532	0.208027	1.498
ses.2:post.:non.exist:wellform.:F0	0.030965	0.749181	0.041
ses.2:post.:non.exist:wellform.:dur	0.666584	0.618206	1.078
ses.2:post.:non.exist:F0:dur	0.159172	0.424167	0.375
ses.2:post.:wellform.:F0:dur	0.346364	0.424167	0.817
ses.2:non.exist:wellform.:F0:dur	-0.226366	0.479570	-0.472
post.:non.exist:wellform.:F0:dur	-0.151607	0.424167	-0.357

ses.2:post.:non.exist:wellform.:int	0.075138	0.539666	0.139
ses.2:post.:non.exist:F0:int	-0.265933	0.388580	-0.684
ses.2:post.:wellform.:F0:int	-0.436816	0.388580	-1.124
ses.2:non.exist:wellform.:F0:int	0.301636	0.441448	0.683
post.:non.exist:wellform.:F0:int	-0.191701	0.388580	-0.493
ses.2:post.:non.exist:dur:int	-0.066294	0.309624	-0.214
ses.2:post.:wellform.:dur:int	-0.108175	0.309624	-0.349
ses.2:non.exist:wellform.:dur:int	-0.774874	0.359135	-2.158
post.:non.exist:wellform.:dur:int	0.231378	0.309624	0.747
ses.2:post.:F0:dur:int	0.230675	0.204801	1.126
ses.2:non.exist:F0:dur:int	0.684640	0.232094	2.950
post.:non.exist:F0:dur:int	0.058764	0.204801	0.287
ses.2:wellform.:F0:dur:int	0.273349	0.229138	1.193
post.:wellform.:F0:dur:int	0.106904	0.204801	0.522
non.exist:wellform.:F0:dur:int	-0.026137	0.417882	-0.063
ses.2:post.:non.exist:wellform.:F0:dur	0.205495	0.848334	0.242
ses.2:post.:non.exist:wellform.:F0:int	-0.079112	0.777161	-0.102
ses.2:post.:non.exist:wellform.:dur:int	0.307802	0.619247	0.497
ses.2:post.:non.exist:F0:dur:int	0.122485	0.409601	0.299
ses.2:post.:wellform.:F0:dur:int	-0.059127	0.409601	-0.144
ses.2:non.exist:wellform.:F0:dur:int	-0.387889	0.466337	-0.832
post.:non.exist:wellform.:F0:dur:int	0.200540	0.409601	0.490
ses.2:post.:non.exist:wellform.:F0:dur:int	1.425371	0.819202	1.740
